# A phase I study of single-agent perifosine for recurrent or refractory pediatric CNS and solid tumors

**DOI:** 10.1371/journal.pone.0178593

**Published:** 2017-06-05

**Authors:** Oren J. Becher, Nathan E. Millard, Shakeel Modak, Brian H. Kushner, Sofia Haque, Ivan Spasojevic, Tanya M. Trippett, Stephen W. Gilheeney, Yasmin Khakoo, David C. Lyden, Kevin C. De Braganca, Jill M. Kolesar, Jason T. Huse, Kim Kramer, Nai-Kong V. Cheung, Ira J. Dunkel

**Affiliations:** 1 Department of Pediatrics, Memorial Sloan Kettering Cancer Center, New York, New York, United States of America; 2 Department of Pediatrics, Northwestern University, Chicago, Illinois, United States of America; 3 Department of Radiology, Memorial Sloan Kettering Cancer Center, New York, New York, United States of America; 4 Department of Radiology, Weill Cornell Medical College, New York, New York, United States of America; 5 Department of Medicine, Duke University Medical Center, Durham, North Carolina, United States of America; 6 Departments of Pediatrics, Weill Cornell Medical College, New York, New York, United States of America; 7 School of Pharmacy, University of Wisconsin, Madison, Wisconsin, United States of America; 8 Department of Pathology, Memorial Sloan Kettering Cancer Center, New York, New York, United States of America; German Cancer Research Center (DKFZ), GERMANY

## Abstract

The PI3K/Akt/mTOR signaling pathway is aberrantly activated in various pediatric tumors. We conducted a phase I study of the Akt inhibitor perifosine in patients with recurrent/refractory pediatric CNS and solid tumors. This was a standard 3+3 open-label dose-escalation study to assess pharmacokinetics, describe toxicities, and identify the MTD for single-agent perifosine. Five dose levels were investigated, ranging from 25 to 125 mg/m2/day for 28 days per cycle. Twenty-three patients (median age 10 years, range 4–18 years) with CNS tumors (DIPG [n = 3], high-grade glioma [n = 5], medulloblastoma [n = 2], ependymoma [n = 3]), neuroblastoma (n = 8), Wilms tumor (n = 1), and Ewing sarcoma (n = 1) were treated. Only one DLT occurred (grade 4 hyperuricemia at dose level 4). The most common grade 3 or 4 toxicity at least possibly related to perifosine was neutropenia (8.7%), with the remaining grade 3 or 4 toxicities (fatigue, hyperglycemia, fever, hyperuricemia, and catheter-related infection) occurring in one patient each. Pharmacokinetics was dose-saturable at doses above 50 mg/m^2^/day with significant inter-patient variability, consistent with findings reported in adult studies. One patient with DIPG (dose level 5) and 4 of 5 patients with high-grade glioma (dose levels 2 and 3) experienced stable disease for two months. Five subjects with neuroblastoma (dose levels 1 through 4) achieved stable disease which was prolonged (≥11 months) in three. No objective responses were noted. In conclusion, the use of perifosine was safe and feasible in patients with recurrent/refractory pediatric CNS and solid tumors. An MTD was not defined by the 5 dose levels investigated. Our RP2D is 50 mg/m2/day.

## Introduction

Aberrant activation of the pathway defined by phosphatidylinositol 3-kinase (PI3K), Akt (protein kinase B), and mammalian target of rapamycin (mTOR) has been observed across a wide range of neoplastic diseases [1–4]. Gene mutations, rearrangements, and amplifications in this pathway lead to disordered control of cell growth and survival and are among the most frequently encountered genetic lesions in human cancers. They are estimated to be present in up to 30% of human malignancies, including pediatric solid and central nervous system (CNS) tumors [5–7]. Evidence suggests that oncogenic alterations in the PI3K/Akt/mTOR signaling cascade are associated with inferior prognoses in many pediatric cancers, including neuroblastoma, rhabdomyosarcoma, high-grade glioma, and medulloblastoma [8–11]. In normal cells, the PI3K/Akt/mTOR network is involved in processes relating to survival, cell growth, angiogenesis, glucose metabolism, and proliferation [4, 12–15]. PI3K signaling is initiated at the cell surface by growth factor receptor tyrosine kinases, activated oncogenic proteins, and/or G-protein-coupled receptors, resulting in complex downstream interactions characterized by crosstalk with other signaling cascades as well as multiple feedback loops. Once activated, PI3K recruits Akt, a serine/threonine protein kinase, to the plasma membrane where it becomes activated via phosphorylation by phosphoinositide-dependent protein kinase 1 (PDK1) and mTOR complex 2 (mTORC2). Phosphorylated Akt in turn activates a number of cellular proteins and inactivates tuberous sclerosis complex 2 (TSC2) resulting in downstream activation of mTOR complex 1 (mTORC1) with a subsequent increase in cell growth, proliferation and survival. Aberrant stimulation of the PI3K/Akt/mTOR pathway can occur via a variety of mechanisms, including activating mutations in Ras, Akt, and receptor tyrosine kinases as well as loss of function of the inhibitory regulator phosphatase and tensin homologue (PTEN) [16–18].

Perifosine (1,1-Dimethyl-4-[[(octadecyloxy)hydroxyphosphinyl]oxy]-piperidinium inner salt) is a third generation orally-available alkylphospholipid with antineoplastic activity. Alkylphospholipids differ from most conventional chemotherapy agents in that they block signal transduction pathways through cell membrane interactions rather than directly damaging DNA in the cell nucleus. The mechanism of action of alkylphospholipids is thought to be primarily mediated through targeting Akt’s pleckstrin-homology domain, resulting in inhibition of Akt membrane localization and phosphorylation. Other potential cytotoxic effects of alkylphospholipids include telomere shortening in cancer cells, induction of autophagy, inhibition of mTOR signaling, activation of cellular stress pathways leading to tumor cell apoptosis, and interference with the synthesis of phospholipids necessary for cell membrane formation, which is often rate-limiting in the setting of hyperproliferation. Of the members of the alkylphospholipid family, perifosine is the best studied and has been demonstrated to be a potent and consistent inhibitor of Akt in pre-clinical and clinical studies. In particular, perifosine has been demonstrated to be cytotoxic in murine models of glioma, medulloblastoma, and neuroblastoma [19–21]. It is further characterized by a long plasma half-life of approximately 4 days and a manageable toxicity profile that differs sufficiently from other chemotherapeutic agents to enable its use in combination regimens [22–33].

## Methods

The primary aim of the study was to determine the MTD of single-agent perifosine in pediatric patients (age ≤ 21 years) with recurrent or refractory pediatric CNS and solid tumors. Secondary aims were to (1) determine whether pharmacokinetic serum levels of perifosine correlate with toxicity, (2) assess preliminary data on the efficacy of perifosine monotherapy, and (3) determine whether molecular features of the tumor were associated with likelihood of response.

**Fig 1:**
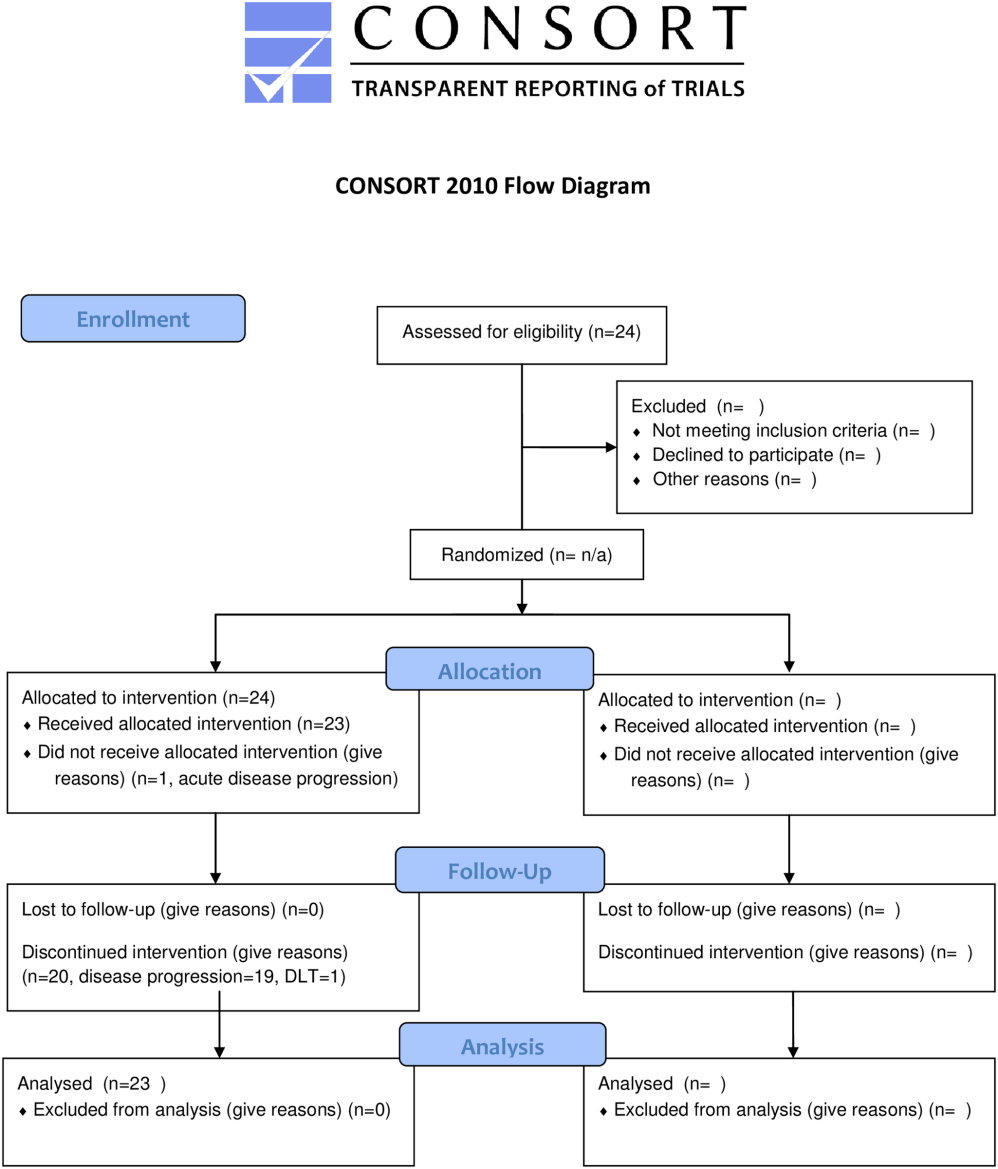
Diagram of study participants.

Patients/guardians consented to the IRB-approved Memorial Sloan Kettering Cancer Center trial #08–091. Twenty-four pediatric patients with recurrent or refractory CNS or solid tumors were enrolled between 2008 and 2014. One enrolled subject never received study prescribed therapy and is not included in this analysis (Fig 1).

Eligibility criteria included: (1) presence of any solid tumor that had failed standard therapy, (2) evidence of tumor by CT, MRI, MIBG scan, serum markers, or tissue sampling, (3) age ≤ 21 years, (4) Karnofsky or Lansky performance status ≥ 50%, (5) adequate organ function [absolute neutrophil count (ANC) ≥ 1000/μL at least 24 hours off filgrastim, platelet count ≥ 75,000/μL at least 1 week post-platelet transfusion, hemoglobin ≥ 8 g/dL at least 1 week post-red blood cell transfusion, aspartate aminotransferase (AST) and alanine aminotransferase (ALT) ≤ 3x the upper limits of normal, total bilirubin ≤ 2 mg/dL, serum creatinine ≤ 1.5x the upper limit of normal for age, or calculated creatinine clearance or nuclear glomerular filtration rate ≥ 70 ml/min/1.73 m^2^], (6) mandated interval since prior therapy [≥ 3 weeks since last non-nitrosourea chemotherapy, ≥ 6 weeks since last nitrosoureas, ≥ 4 weeks since last radiation therapy], (7) ability to swallow tablets whole, and (8) agreement to practice adequate contraception and not breastfeed. Exclusion criteria included (1) pregnancy, (2) uncontrolled active infection or serious medical illness, (3) use of combination anti-retroviral therapy in patients who are known to be HIV-positive, and (4) enzyme-inducing anti-convulsant usage.

### Treatment protocol

This was a standard 3+3 phase I dose escalation study (NCT00776867) investigating 5 dose levels. Perifosine was only available as 50-mg tablets, complicating the phase I dose escalation scheme. A loading dose was administered on day 1 and subsequent maintenance doses were administered every 1 to 4 days, depending on dose level and BSA, with the goal of achieving dose levels of 25, 50, 75, 100, and 125 mg/m^2^/day (Tables 1–5). This dosing scheme, incorporating drug administration as infrequently as every 4 days, was made possible by perifosine’s long half-life. Treatment was continued until disease progression, intolerable toxicity, DLT, or death was encountered. Subjects experiencing DLT but with evidence of clinical benefit at dose level 1, 2, or 3 were eligible to remain on study with a dose level reduction.

**Table 1:** Perifosine dose level 1 (~25 mg/m^2^/day).

BSA (m^2^)	Loading dose day 1	Maintenance dose—starting on day 2
0.4 to 0.59	50mg	50mg every four days
0.6 to 0.79	50mg	50mg every three days
0.8 to 1.2	100mg	50mg every other day
1.21 to 1.6	150mg	50mg daily five days per week
> 1.6	150mg	50mg daily

**Table 2:** Perifosine dose level 2 (~50 mg/m^2^/day).

BSA (m^2^)	Loading dose day 1	Maintenance dose—starting on day 2
0.4 to 0.59	100mg	50mg every other day
0.6 to 0.79	100mg	50 mg daily five days per week
0.8 to 1.2	100mg BID	50mg daily
1.21 to 1.6	150mg BID	100mg daily five days per week
> 1.6	150mg BID	100mg daily

**Table 3:** Perifosine dose level 3 (~75 mg/m^2^/day).

BSA (m^2^)	Loading dose day 1	Maintenance dose—starting on day 2
0.4 to 0.59	100mg	50mg daily five days per week
0.6 to 0.79	100mg	50mg daily
0.8 to 1.2	100mg BID	50mg daily alternating with 100mg daily
1.21 to 1.6	150mg BID	100mg daily
> 1.6	150mg BID	100mg daily alternating with 150mg daily

**Table 4:** Perifosine dose level 4 (~100 mg/m^2^/day).

BSA (m^2^)	Loading dose day 1	Maintenance dose—starting on day 2
0.4 to 0.59	100mg	50mg daily
0.6 to 0.79	100mg	50mg daily alternating with 100mg daily
0.8 to 1.2	100mg BID	100mg daily
1.21 to 1.6	150mg BID	150mg daily six days per week and 100mg daily for one day per week
> 1.6	150mg BID	150mg daily alternating with 200mg daily

**Table 5:** Perifosine dose level 5 (~125 mg/m^2^/day).

BSA (m^2^)	Loading dose day 1	Maintenance dose—starting on day 2
0.4 to 0.59	100mg	50mg daily five days per week and 100mg daily two days per week
0.6 to 0.79	100mg	100mg daily five days per week and 50mg daily two days per week
0.8 to 1.2	100mg BID	100mg daily alternating with 150mg daily
1.21 to 1.6	150mg BID	150mg daily alternating with 200mg daily
> 1.6	150mg BID	200mg daily

Subjects were seen for physical examination and laboratory assessment (complete blood count, serum chemistry) prior to study enrollment, approximately 1 week after starting treatment, and then during the first week of every 28-day treatment cycle. Tumor assessments were performed approximately every 8 weeks.

Toxicity was assessed according to the Common Toxicity Criteria (version 3.0) of the National Cancer Institute, National Institutes of Health. DLT was defined as (1) any non-hematological toxicity grade ≥ 3 [except for grade 3 nausea, vomiting, and diarrhea that could be controlled within 24 hours with supportive care measures]; (2) grade 4 neutropenia on 2 consecutive blood counts drawn at least 72 hours apart; (3) grade 4 febrile neutropenia [ANC < 1000/μL and fever ≥ 38.5°C] or grade ≥ 3 documented infection with ANC < 1000/μL, or; (4) a platelet count < 25,000/μL. Grade ≥ 3 decrease in hemoglobin that could be corrected to at least 8 g/dl (grade 2) by transfusion of red blood cells, grade ≥ 3 leukopenia in the absence of dose-limiting neutropenia, and grade ≥ 3 lymphopenia were not considered DLTs.

### Correlative studies

Samples for pharmacokinetic analysis were obtained at baseline and during weeks 2 through 4 of cycle 1. At each time point, heparinized blood was collected into a plastic vacutainer to minimize adhesion of perifosine. Plasma was separated by centrifugation and stored in polypropylene cryovials at -70°C until assayed. Perifosine in plasma was measured by a validated reversed phase liquid chromatography/electrospray mass spectrometry method as developed and validated by Woo *et al*. [34].

Tumor tissue: Fifteen unstained paraffin slides and/or 100 mg of flash frozen tissue if available were obtained from the surgery closest to initiation of this clinical trial. These were used to evaluate molecular markers that may predict sensitivity to perifosine, including PI3K/Akt pathway activity as measured by phosphorylation of both Akt (AKT) and the proline-rich Akt substrate of 40 kDa (PRAS40) as well as proliferation rate as assessed by MIB-1 immunostaining.

### Response criteria

For subjects with tumors other than neuroblastoma, responses were assessed by the study radiologist and principal investigator (SH, ID) via the Response Evaluation Criteria in Solid Tumors (RECIST) [35]. For subjects with neuroblastoma, responses were assessed by a single investigator (BK) using the International Neuroblastoma Response Criteria (INRC) [36].

### Statistics

The DLT assessment period was the first 28-day cycle. If therapy was discontinued during the first cycle for reasons other than toxicity, an additional subject could be enrolled at that dose level to ensure adequate evaluation of toxicity. No intra-patient dose escalation was permitted.

## Results

### Clinical

Twenty-three subjects (median age 10 years, range 4 to 18 years) received a total of 189 cycles. Nine (39%) were male and 14 female. Thirteen subjects had CNS tumors (diffuse intrinsic pontine glioma [DIPG] n = 3, high-grade glioma n = 5, medulloblastoma n = 2, ependymoma n = 3), 8 had neuroblastoma, 1 had Wilms tumor, and 1 had Ewing sarcoma/primitive neuroectodermal tumor (PNET) (Table 6). A median of 3 cycles were initiated and a median of 2 cycles were completed per patient, ranging from <1 to 68 cycles.

**Table 6:** Patient characteristics and responses.

Patient	Dose Level	Age	Sex	Diagnosis	Prior RT	Number of Prior Chemotherapy Regimens	Best Response (Duration)	p-AKT	p-PRAS40	MIB-1
1	1	18	M	medulloblastoma	yes	8	PD			
2	1	17	M	medulloblastoma	yes	5	PD	++	++	30%
3	1	10	M	neuroblastoma	yes	6	NR (11 months)	-	-	<2%
4	2	13	F	glioblastoma	yes	1	PD			
5	2	12	F	neuroblastoma	yes	7	NR (63 months)	-	-	<1%
6	2	17	M	anaplastic astrocytoma	yes	2	SD (2 months)			
7	3	9	F	glioblastoma	yes	3	SD (2 months)			
8	3	5	F	anaplastic astrocytoma	yes	3	SD (2 months)	-	-	10%
9	3	14	F	anaplastic astrocytoma	yes	1	SD (2 months)	-	-	10%
10	3	13	F	ependymoma	yes	7	PD	-	++	10%
11	3	4	F	neuroblastoma	no	5	NR (2 months)			
12	4	8	M	neuroblastoma	yes	10	PD			
13	4	18	F	Ewing sarcoma/PNET	yes	5	PD	-	-	60%
14	4	13	M	neuroblastoma	yes	9	NR (4 months)			
15	4	7	M	neuroblastoma	yes	5	NR (42 months)	-	-	20%
16	4	7	M	neuroblastoma	yes	4	PD	-	+	70%
17	4	5	F	DIPG	yes	0	PD			
18	5	16	F	Wilms tumor	yes	6	PD	-	+	70%
19	5	6	M	DIPG	yes	1	PD			
20	5	9	F	DIPG	yes	0	SD (2 months)			
21	5	16	F	ependymoma	yes	2	PD	+	+	30%
22	5	7	F	ependymoma	yes	6	PD	-	+	90%
23	5	7	F	neuroblastoma	yes	4	PD	++	+++	90%

RT = radiation therapy, DIPG = diffuse intrinsic pontine glioma, PNET = primitive neuroectodermal tumor, F = female, M = male, NR = no response per INRC, PD = progressive disease per RECIST or INRC, SD = stable disease per RECIST, p-AKT = phosphorylated Akt, p-PRAS40 = phosphorylated proline-rich Akt substrate of 40 kD

### Toxicity

Perifosine was generally well tolerated. The most common toxicities of any grade (at least possibly related) were fatigue (65.2%), nausea (65.2%), hyperglycemia (60.9%), vomiting (56.5%), and decreased leukocytes (47.8%), with the vast majority of these toxicities ≤ grade 2. Only one DLT occurred (grade 4 hyperuricemia at dose level 4). The most common grade 3 or 4 toxicity at least possibly related to perifosine was neutropenia (8.7%), with the remaining grade 3 or 4 at least possibly related toxicities (fatigue, hyperglycemia, fever, hyperuricemia, and catheter-related infection) occurring in one patient each. Table 7 summarizes toxicities that were considered at least possibly related and observed in more than 10% of subjects or ≥ grade 3 (even if seen in fewer than 10% of subjects). Although the initial study design only included three dose levels of perifosine, the lack of DLT at the first 3 dose levels prompted the addition of dose levels 4 and 5 as permitted by protocol amendment. Enrollment on dose level 3 was concluded when this protocol amendment was approved, following enrollment of the fifth patient. The second patient (diagnosed with Ewing sarcoma/PNET) enrolled at dose level 4 experienced a grade 4 elevation in uric acid, considered to be possibly related to perifosine. That patient was therefore removed from the protocol and the dose level 4 cohort was expanded to six patients. No further grade ≥ 3 toxicities were observed in any other patient treated at dose level 4 and an additional 3 patients were enrolled at dose level 5. As dose level 5 was under consideration as the MTD, 3 final patients were enrolled at that dose level to confirm lack of toxicity. One patient at dose level 5 experienced a grade 3 hyperglycemia (possibly related) on the day of protocol removal for disease progression. There were no other grade ≥ 3 toxicities or DLTs in the patients treated at dose level 5.

**Table 7:** Toxicity summary.

	Any Grade		Grade 3 or 4	
Hematologic Adverse Events	No.	%	No.	%
Decreased leukocytes	11	47.83%	0	0.00%
Decreased hemoglobin	10	43.48%	0	0.00%
Decreased neutrophils	6	26.09%	2	8.70%
Decreased platelets	5	21.74%	0	0.00%
Lymphopenia	4	17.39%	0	0.00%
**Non-hematologic Adverse Events**				
Fatigue	15	65.22%	1	4.35%
Nausea	15	65.22%	0	0.00%
Hyperglycemia	14	60.87%	1	4.35%
Vomiting	13	56.52%	0	0.00%
Diarrhea	10	43.48%	0	0.00%
ALT	9	39.13%	0	0.00%
Anorexia	7	30.43%	0	0.00%
AST	6	26.09%	0	0.00%
Fever	5	21.74%	1	4.35%
Hypocalcemia	5	21.74%	0	0.00%
Hypokalemia	5	21.74%	0	0.00%
Hyponatremia	5	21.74%	0	0.00%
Pain—abdomen NOS	5	21.74%	0	0.00%
Infection URI	4	17.39%	0	0.00%
Constipation	3	13.04%	0	0.00%
Flatulence	3	13.04%	0	0.00%
Hypomagnesemia	3	13.04%	0	0.00%
Hypophosphatemia	3	13.04%	0	0.00%
Pain—stomach	3	13.04%	0	0.00%
Hyperuricemia	1	4.35%	1	4.35%
Infection—catheter related	1	4.35%	1	4.35%

Notes: Toxicities considered to be at least possibly related. Non-hematological toxicities seen in either > 10% of subjects or ≥ grade 3.

### Responses

Response results are detailed in Table 6. Among 4 of 5 subjects with recurrent high-grade astrocytoma, the best responses were stable disease for 2 months in each. Three subjects with recurrent DIPG were treated with stable disease in 1 for 2 months and progressive disease in 2. All patients with ependymoma, medulloblastoma, Wilms tumor, and Ewing sarcoma/PNET had progressive disease as their best response. Five of 8 subjects with neuroblastoma experienced disease stability (labeled “no response” in the nomenclature of the INRC and defined as a less than 50% reduction of some or all measurable lesions, but no increase of greater than 25% in these lesions and no new lesions) for 2, 4, 11, 42, and 63 months, respectively. Three additional neuroblastoma patients had progressive disease, defined as a greater than 25% increase in any preexisting lesion or any new lesion.

### Pharmacokinetics

**Fig 2:**
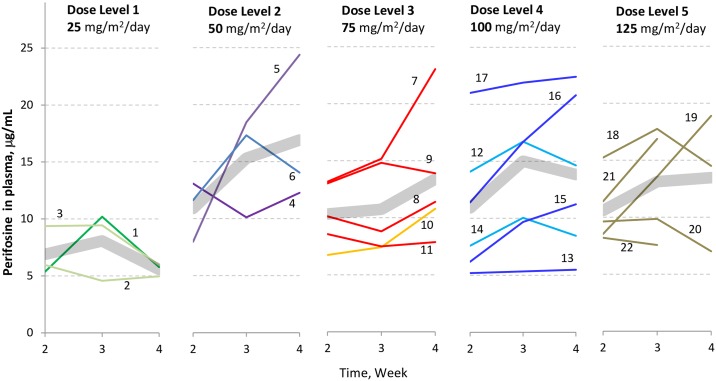
Steady state plasma concentration of perifosine at weeks 2, 3, and 4. Concentrations were plotted for each study subject whose samples were available for calculation (patients 1–22) across the dose groups; enumerated individual subject trace; grey, semi-transparent thick line represents the arithmetic mean of the plasma concentration at the corresponding time-point for the given dose group. Saturable dose exposure above 50 mg/m^2^/day dose is evident albeit large inter-patient variability, consistent with findings reported in previous adult studies.

**Fig 3:**
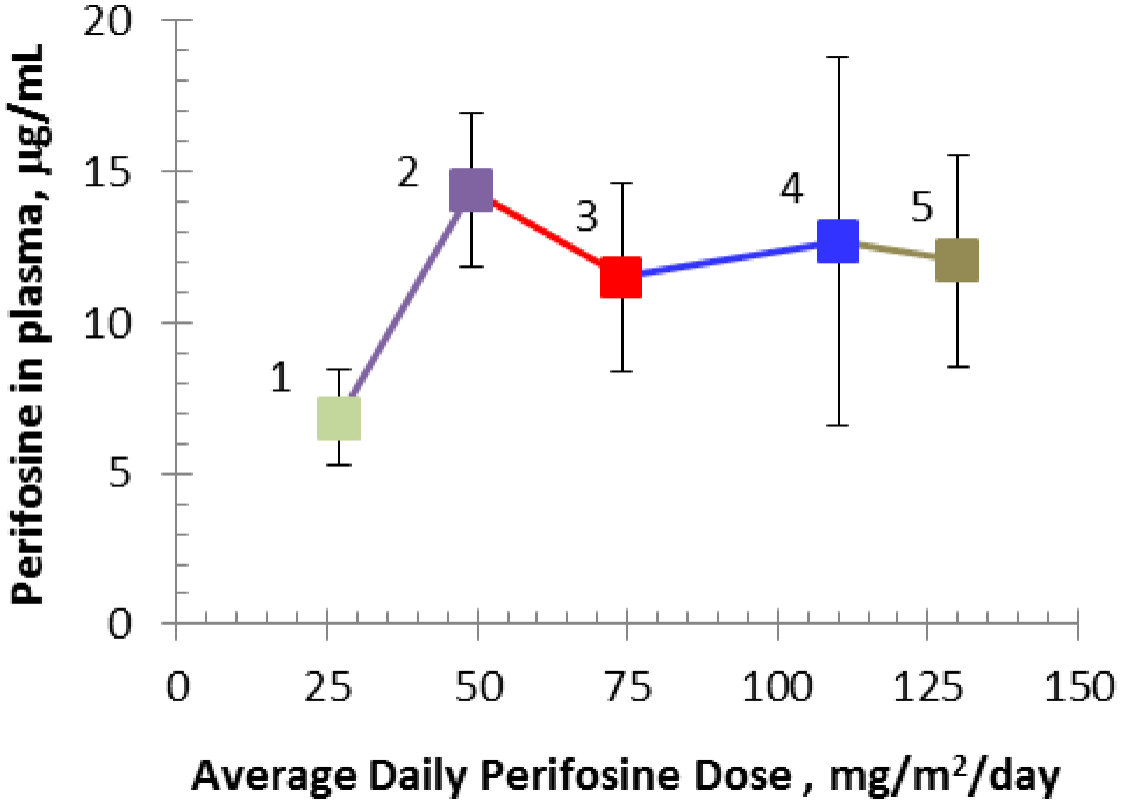
Correlation plot of perifosine steady state plasma concentration versus daily dose (actually administered). Error bars represent single standard deviation; enumerated dose groups. Saturable dose exposure above 50 mg/m^2^/day is observed in pediatric patients.

The BSA grouping-based dosing scheme (Tables 1–5) was generally successful in approximating the target perifosine doses using 50-mg tablets (Table 8). Pharmacokinetic evaluation was possible with samples from 22 patients. Individual steady state plasma concentrations found are presented in Fig 2. Rather wide interpatient variability is observed in accordance with results from published adult studies [32,37]. Appreciable intra-patient variability can also be inferred in some subjects (notably patients 5, 7, 16 and 19). Average steady state levels of perifosine were calculated for each dose level (Table 8 and Fig 3). We observed saturable dose exposure at doses above 50 mg/m^2^/day.

**Table 8:** Perifosine pharmacokinetics.

Perifosine Dose Level	Patients	Target Perifosine Dose—mg/m^2^/day	Given Perifosine Dose (standard deviation)—mg/m^2^/day	Average Plasma Level (standard deviation)—μg/mL; μM
1	1,2,3	25	27 (1.3)	6.8 (1.6); 18.4 (4.3)
2	4,5,6	50	49 (2.4)	14.4 (2.5); 39.0 (6.8)
3	7,8,9,10,11	75	74 (5.6)	11.5 (3.1); 31.1 (8.5)
4	12,13,14,15,16,17	100	110 (9.1)	12.7 (6.1); 34.4 (16.5)
5	18,19,20,21,22	125	130 (13.1)	12.1 (3.5); 32.8 (9.5)

### Tumor tissue biological assays

Biological assays were performed on 13 available tumors (Table 6). Using p-PRAS40 as a surrogate for Akt activation, we tested whether Akt activation correlated with SD/PR in this small patient cohort. Of the 13 tumors with available information regarding Akt activation status, 7 demonstrated Akt activation while 6 tumors did not. Of the 6 tumors without Akt activation, 5 had SD/NR while one had PD. Of the 7 tumors demonstrating Akt activation, all had PD. Using the Fisher’s exact test, the 2-sided p-value was 0.0047, demonstrating that lack of Akt activation is significantly associated with response.

## Discussion

This phase I study represents the first evaluation of the safety and tolerability of perifosine monotherapy in pediatric patients with recurrent or refractory CNS and solid tumors. Our BSA grouping-based dosing schedule was successful in approximating perifosine doses using 50-mg tablets. As perifosine was generally well-tolerated at all investigated dose levels, an MTD was not defined. Similarly, lack of toxicity made the secondary study aim of investigating the relationship between serum levels of perifosine and toxicity less relevant. Our steady state perifosine plasma levels show saturable dose exposure (presumably due to saturable drug absorption) as observed in adult studies but were generally somewhat higher in our pediatric patients. This supports our observation that the “plateau” in pediatric patients is reached at lower maintenance doses (at 50 mg/m^2^/day), whereas in 2 adult studies the dose exposure plateau was reached at doses higher than 100 mg/m^2^/day [32, 37]. Additionally, several clinical trials have observed saturable dose-exposure to perifosine, with increasing toxicity (particularly gastrointestinal toxicity and fatigue) but no improvement in efficacy at higher doses [31, 37, 38]. The achieved plasma levels in this study compare favorably to the IC50 values published for preclinical pediatric tumor models. A wide range of IC50 values have been reported with perifosine, ranging from as low as 10 μM to as high as 30 μM in different neuroblastoma cell lines, for example [21]. Rhabdomyosarcoma cell lines were relatively sensitive to perifosine, with IC50 values around 10 μM, while medulloblastoma cell lines were comparatively more resistant, with IC50 values of 25 μM [7, 39]. Given our observation of saturable dose exposure at doses above 50 mg/m2/day that would be expected to result in anti-tumor activity, our recommended Phase 2 dose is 50 mg/m2/day.

Although we observed no objective responses within our patient cohort, a number of patients experienced disease stabilization. This is consistent with results published from previous early phase clinical trials of perifosine monotherapy in adults as well as the Children’s Oncology Group’s phase I trial of single-agent MK-2206, another Akt inhibitor, in children, which suggested that Akt inhibition resulted in anti-proliferative rather than tumoricidal effects [40, 41]. The lack of objective responses suggests that this class of agents may be more effective in combination with additional cytotoxic and/or targeted agents in future studies. For example, in early phase clinical trials, the use of Akt inhibitor-containing combination regimens in advanced adult cancers has resulted in complete or partial responses [42–44]. However, two subsequent phase 3 studies of combination therapy with perifosine in adults with refractory colorectal cancer or multiple myeloma did not demonstrate a survival benefit, prompting Aeterna Zentaris to discontinue those trials [45, 46]. Our observation that tumors with Akt activation were significantly less likely to have SD/NR and significantly more likely to have PD in response to perifosine is in concordance with the published literature which shows Akt activation to be a poor prognostic factor in a variety of pediatric tumor types. Tumors with Akt activation are more aggressive and less likely to respond to any experimental agent, particularly in the setting of recurrent disease. In support of this, the average MIB-1 of p-PRAS40 positive tumors in our cohort was 55.7% ± 32.1 while that of p-PRAS40 negative tumors was 17.2% ± 22.1. Additionally, perifosine is not a typical Akt inhibitor like MK-2206, an allosteric Akt inhibitor. As an alkylphospholipid, it has several additional proposed mechanisms of action in addition to its activity as an inhibitor of Akt. For example, it has recently been reported to inhibit telomerase activity as low micromolar doses [25]. Perifosine’s effect on telomerase activity may or may not be related to its effect on Akt activation, as Akt has been previously shown to form a complex with the protein subunit of telomerase [47]. Other putative mechanisms include activation of the stress-activated protein kinase/c-Jun NH2 terminal kinase (SAPK/JNK pathway), also at low micromolar levels, although this may be related to inhibition of Akt [48]. In addition, perifosine has also been shown to degrade components of the mTOR pathway and to induce autophagy at doses as low as 5 μM [49]. Based on our PK data with steady state plasma levels of perifosine above 10 μM observed in most patients, all of these additional mechanisms of action could have been at play in our patient cohort, including perifosine’s autophagy-promoting activity which may have resulted in diminished anti-tumor activity.

There is preclinical evidence to suggest that subsets of patients may be more likely to respond to Akt inhibitors based the presence or absence of specific genetic alternations. For example, cell lines with PTEN loss or *PIK3CA* mutations have been shown to be significantly more sensitive to the Akt inhibitors in a variety of tumor types, while cells lines with *RAS* mutations are generally resistant even in the presence of concomitant *PIK3CA* mutations [42–44, 50]. Consideration of PI3K/Akt/mTOR pathway activation status would therefore be expected to aid in the selection of patients who would be more likely to respond to Akt inhibition. Further, a recent study of perifosine in T-cell acute lymphoblastic leukemia cell lines with constitutive activation of the PI3K/Akt/mTOR pathway showed that the combination of perifosine with two additional small molecule inhibitors of Akt resulted in synergism for the induction of cell cycle arrest as well as of apoptosis and autophagy, suggesting that multi-inhibition treatment against Akt may be a potentially advantageous pharmacologic treatment strategy in tumors with aberrant PI3K/Akt/mTOR pathway activation [51].

Prolonged disease stability in a subset of neuroblastoma patients treated at the first three dose levels prompted the creation of an expansion cohort of an additional 14 neuroblastoma patients, the results of which are reported separately [52]. One possible explanation for the lack of objective response in patients with brain tumors may relate to CNS penetration. A recent evaluation of cerebrospinal fluid pharmacokinetics in adult non-tumor bearing rhesus monkeys showed that the CNS penetration of oral perifosine was limited [53]. However, the presence of a brain tumor is known to alter drug penetration through the blood brain barrier, generally rendering it more permeable as compared with the normal brain vasculature, albeit within a spectrum of barrier integrity that may be influenced by specific CNS tumor type [54–56]. Future studies of Akt inhibitors in brain tumor patients should include assessment of whether these drugs are capable of crossing the blood brain barrier in sufficient concentrations to target CNS neoplasms. Additionally, strategies to bypass the blood brain barrier are under investigation. A recent study of convection enhanced delivery (CED) of perifosine in a mouse brainstem glioma model showed no serious acute toxicities at doses demonstrated to be effective in cell culture [57]. Further studies are needed to determine the long-term safety and potential clinical application of this drug delivery technique.

In conclusion, perifosine in pediatric patients at the dosing schedule employed is safe and well-tolerated. Evaluation of the optimal method of drug delivery in the case of CNS tumors, the rational combination of perifosine with cytotoxic chemotherapy, alternative Akt inhibitors or other targeted agents, and the potential selection of patient subsets that can be predicted to respond to Akt pathway inhibition based on molecular characteristics remain to be established.

## Supporting information

S1 File TREND checklist. (PDF)

S2 File Study protocol MSKCC 08–091 A12 (final version). (PDF)
