# Supplementary material for: A phase I study of single-agent perifosine for recurrent or refractory pediatric CNS and solid tumors
Source: PLoS One. 2017 Jun 5;12(6):e0178593. doi: 10.1371/journal.pone.0178593 (PMC5459446; doi:10.1371/journal.pone.0178593)
Supplement: S2 File — (PDF) [file pone.0178593.s002.pdf]

Memorial Sloan-Kettering Cancer Center
IRB Protocol

IRB#: 08-091 A(12)

Phase I Study of Single Agent Perifosine for Recurrent Pediatric Solid Tumors

MSKCC THERAPEUTIC/DIAGNOSTIC PROTOCOL

**Principal Investigator/Department:**	Ira Dunkel, MD	Pediatrics
**Co-Principal Investigator(s)/Department:**	Tanya Trippett, MD	Pediatrics
	Stephen Gilheeney MD, MMS	Pediatrics
**Investigator(s)/Department:**	Yasmin Khakoo, MD	Pediatrics
	David Lyden, MD, PhD	Pediatrics
	Shakeel Modak, MD	Pediatrics
	Mary Petriccione, CPNP	Pediatrics - Nursing
	Brian Kushner, MD	Pediatrics
	Nai-Kong Cheung MD, PhD	Pediatrics
	Kim Kramer, MD	Pediatrics
	Cheryl Fischer, CPNP	Pediatrics - Nursing
	Camelia Sima, MD, MS	Epidemiology-Biostatistics
	Sofia Haque, MD	Radiology
	Kevin De Braganca, MD	Pediatrics
	Nathan Millard, MD	Pediatrics
	Ellen Basu, MD, PhD	Pediatrics
	Venkatraman Seshan, PhD	Epidemiology-Biostatistics
	Maria Donzelli, CPNP	Pediatrics - Nursing
	Stephen Roberts, MD	Pediatrics
	Jason Huse, MD	Pathology

Memorial Sloan-Kettering Cancer Center
1275 York Ave.
New York, NY 10021

IRB PB **Amended: 8/13/14**

**Consenting Professional(s)/Department:**	Shakeel Modak MD	Pediatrics
	Yasmin Khakoo, MD	Pediatrics
	Stephen Gilheeney, MD, MMS	Pediatrics
	David Lyden, MD, PhD	Pediatrics
	Ira Dunkel, MD	Pediatrics
	Tanya Trippett, MD	Pediatrics
	Ellen Basu, MD	Pediatrics
	Nai-Kong Cheung MD, PhD	Pediatrics
	Kim Kramer, MD	Pediatrics
	Brian Kushner, MD	Pediatrics
	Stephen Roberts, MD	Pediatrics
	Kevin De Braganca, MD	Pediatrics

Collaborating Institution(s):

Jill M. Kolesar, Pharm. D.
University of Wisconsin
Role: Specimen Analysis

Oren Becher, MD
Duke University Medical Center
Role: Data Analysis

Table of Contents

MSKCC THERAPEUTIC/DIAGNOSTIC PROTOCOL ... 1
1.0 PROTOCOL SUMMARY AND/OR SCHEMA ... 5
2.0 OBJECTIVES AND SCIENTIFIC AIMS ... 5
3.0 BACKGROUND AND RATIONALE ... 5
3.1 RECURRENT PEDIATRIC TUMORS ... 5
3.2 PERIFOSINE (NSC 639966, D21266, KRX-0401) ... 6
3.3 PERIFOSINE MECHANISM OF ACTION ... 6
3.4 PRE-CLINICAL STUDIES ... 7
3.5 PERIFOSINE CLINICAL DATA ... 11
3.6 POTENTIAL IMPACT ON METABOLISM BY HEPATIC ENZYME INDUCING ANTIEPILEPTIC DRUGS (EIAEDS) ... 19
3.7 PHASE I EXPERIENCE IN CHILDREN ... 20
4.0 OVERVIEW OF STUDY DESIGN/INTERVENTION ... 20
4.1 DESIGN ... 20
4.2 INTERVENTION ... 20
5.0 THERAPEUTIC/DIAGNOSTIC AGENTS ... 20
6.0 CRITERIA FOR SUBJECT ELIGIBILITY ... 21
6.1 SUBJECT INCLUSION CRITERIA ... 21
6.2 SUBJECT EXCLUSION CRITERIA ... 22
7.0 RECRUITMENT PLAN ... 22
8.0 PRETREATMENT EVALUATION ... 22
8.1 PRETREATMENT EVALUATION FOR PERIFOSINE MONOTHERAPY ... 22
9.0 TREATMENT/INTERVENTION PLAN ... 23
9.1 DOSING- PERIFOSINE MONOTHERAPY ... 23
9.2 PHARMACOKINETICS (ALL PATIENTS) ... 26
9.3 DEFINITION OF DOSE-LIMITING TOXICITY (DLT) AND TREATMENT MODIFICATIONS ... 28
9.4 SUPPORTIVE CARE GUIDELINES (ALL PATIENTS) ... 29
10.0 EVALUATION DURING TREATMENT/INTERVENTION ... 31
10.1 CLINICAL ... 31
10.2 LABORATORY ... 31
10.3 IMAGING STUDIES ... 31
10.4 BIOLOGICAL ASSAY ... 31
11.0 TOXICITIES/SIDE EFFECTS ... 33
12.0 CRITERIA FOR THERAPEUTIC RESPONSE/OUTCOME ASSESSMENT ... 35
13.0 CRITERIA FOR REMOVAL FROM STUDY ... 36
14.0 BIOSTATISTICS ... 36
15.0 RESEARCH PARTICIPANT REGISTRATION AND RANDOMIZATION PROCEDURES ... 38
15.1 RESEARCH PARTICIPANT REGISTRATION ... 38
16.0 DATA MANAGEMENT ISSUES ... 39

16.1 QUALITY ASSURANCE 39
16.2 DATA AND SAFETY MONITORING 39
17.0 PROTECTION OF HUMAN SUBJECTS 40
17.2 SERIOUS ADVERSE EVENT (SAE) REPORTING 40
18.0 INFORMED CONSENT PROCEDURES 42
19.0 REFERENCE(S) 44
20.0 APPENDICES 47
20.1 KARNOFSKY PERFORMANCE STATUS 48
20.2 LANSKY SCORE (FOR CHILDREN) 49
20.3 SUPPORTIVE CARE GUIDELINES FOR CONTROLLING SIDE EFFECTS ASSOCIATED WITH THE ADMINISTRATION OF PERIFOSINE 50

1.0 PROTOCOL SUMMARY AND/OR SCHEMA

Open label, phase I study of oral perifosine in recurrent/progressive pediatric solid tumors. This will be a dose escalation study to determine the maximum tolerated dose (MTD) of perifosine alone in recurrent/progressive pediatric tumors. A standard 3+3 dose escalation design will be employed with 3-6 patients at each dose level. All patients will receive perifosine at a loading dose on the first day, followed by a maintenance dose to start on day two until progression as specified in section 9.1 where each patient will be assigned to a dosing group based on one's body surface area (BSA).

2.0 OBJECTIVES AND SCIENTIFIC AIMS

A. Primary Objectives: Safety
- Maximum Tolerated Dose (MTD) of perifosine monotherapy in children with cancer

B. Secondary Objectives:
- To determine whether pharmacokinetic serum levels correlate with toxicity
- If previously resected tissue is available, determine whether molecular features predict response including
  - Elevated PI3K/AKT/mTOR signaling
  - Elevated RAS/MAPK signaling
  - Cell cycle markers

3.0 BACKGROUND AND RATIONALE

3.1 Recurrent pediatric Tumors

Approximately 12,400 children and adolescents younger than 20 years of age are diagnosed with some type of cancer each year. Among these, approximately 2,300 children and adolescents die of cancer each year making cancer the most common cause of disease-related mortality for children 1 to 19 years of age.[1] There are 12 major types of childhood cancers, and of these leukemias and central nervous system tumors (ie, gliomas and medulloblastomas) account for over 50% of the new cases. The remainder of new cases include extracranial tumors such as neuroblastomas, Wilms tumors, and rhabdomyosarcomas.[2]

From 1975 through 1998, the incidence of children diagnosed with all forms of invasive cancer increased from 11.4 cases per 100,000 children to 15.2 cases per 100,000 children.[3] Although improvements in imaging equipment and treatments have resulted in an overall decline in mortality and increased survival, approximately half of all children with malignant solid tumors will experience relapse and, of these children, less than 10% will achieve long-term survival after standard multimodal therapy.[3,4]

3.2 Perifosine (NSC 639966, D21266, KRX-0401)

Alkylphospholipids represent a new class of lipid-related compounds that exhibit promising anticancer activity and a different spectrum of toxicity than conventional cytotoxic agents. Perifosine (1,1-dimethyl-4 [[(octadecyloxy) hydroxyphosphinyl]oxy]-piperidinium inner salt) is a synthetic, substituted heterocyclic alkylphospholipid, structurally related to miltefosine (NSC 60558, D-18506). The anti-tumor activity of miltefosine was initially evaluated in the 1980s, and it is licensed in Europe as a topical application for the treatment of patients with cutaneous metastases from breast cancer. It is also used in an oral formulation to treat leishmaniasis. Because the only major toxicities of miltefosine are gastrointestinal --and this was thought be a local rather than a central effect of the drug, numerous analogues were developed to see if a less toxic analogue could be identified. Perifosine was identified as a potentially active and better tolerated analog of miltefosine. Its spectrum of activity across the NCI 60 cell line screen was very similar to miltefosine (Pearson correlation coefficient = 0.817). Both miltefosine and perifosine had very unique patterns of *in vitro* cell growth inhibition that are distinctly different from most cytotoxic agents. Perifosine has been shown to be more active and better tolerated than miltefosine in preclinical models [5]. Perifosine exhibited marked activity in animal and human tumor cell lines resistant to standard chemotherapeutic agents with relative sparing of normal cells, including macrophages, bone marrow cells and normal glial cells in a glioblastoma model.

Alkylphospholipids are known to affect tumor cell proliferation, differentiation, invasion, and metastasis. These compounds are absorbed directly into cell membranes where they accumulate (reviewed in [6]). Although there is considerable evidence that the plasma membrane is the primary site of action, alkylphospholipids may be widely distributed throughout the cytoplasm and possibly within the nucleus. [7, 8]

3.3 Perifosine Mechanism of Action

Perifosine has been shown to inhibit AKT activation *in vitro* and *in vivo* in mouse models. This is of interest because this pathway is known to be overactive in a large number of pediatric solid tumors. As an example of this pathway’s central role in oncogenesis, combined activation of AKT pathway with Ras pathway activation induces GBMs in neonatal mice [9] mimicking pediatric GBMs while neither AKT pathway activation nor RAS pathway alone is sufficient.

Figure 1: AKT cascade is inhibited by perifosine (courtesy Andrew Lassman).

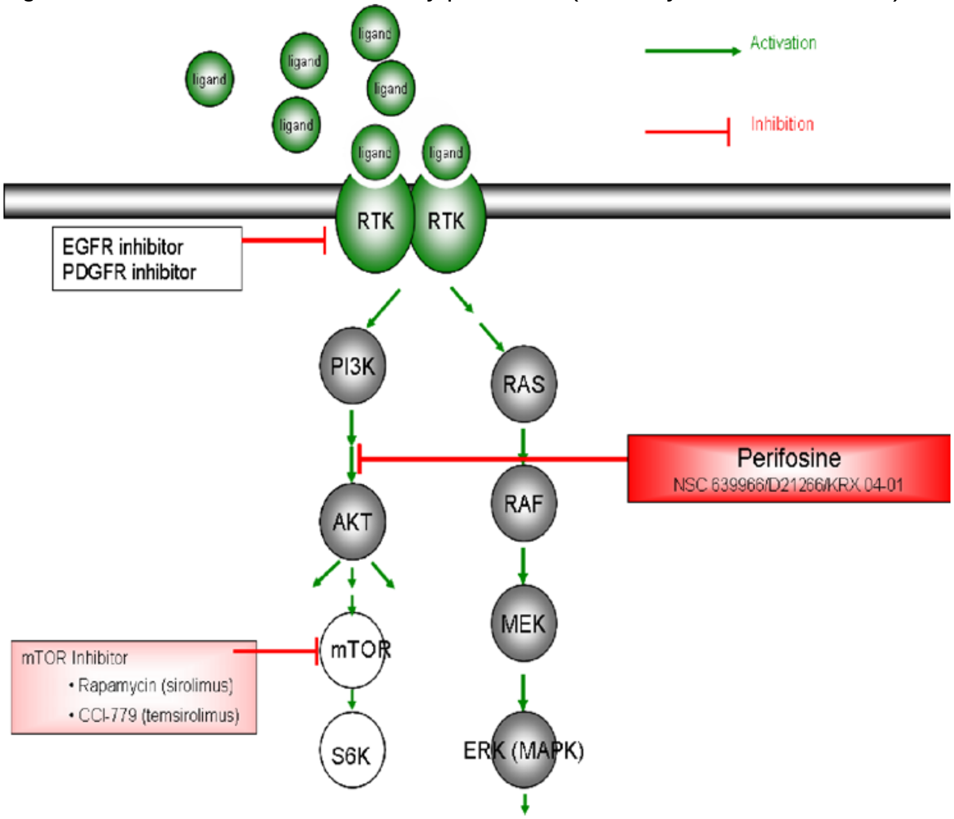

Aberrant activation of the phosphatidylinositol 3'-kinase (PI3K)/AKT pathway, a key survival cascade has been associated with poor prognosis or proliferation in several pediatric tumors such as neuroblastoma, glioblastoma, rhabdomyosarcoma, and medulloblastoma [10-13].

Furthermore, inhibition of AKT pathway with perifosine in genetic engineered murine models of medulloblastomas and gliomas (Holland lab) results in regional necrosis and increased apoptosis (Hambardzumyan et al., in preparation) Other direct inhibitors of AKT have been too toxic for clinical use. By contrast, perifosine has been used with limited toxicity in adults with some efficacy in distinct histological subtypes (below). This will be the first study of perifosine in pediatrics.

3.4 Pre-clinical studies

In vitro

Prior to stimulation of the PI3K/AKT pathway, AKT is localized in the cellular cytoplasm. Following stimulation and activation of PI3K, AKT is recruited to the cell membrane, where it then localizes. Since the long alkyl chain of the alkylphosphocholines can insert into the outer leaflet of the plasma membrane and since these agents use lipids, such as those found in cellular

membranes, as substrates, it seemed plausible that perifosine might inhibit a transduction pathway component known to be dependent for its activity on membrane localization.

Investigators at the US National Cancer Institute (NCI) first studied the effects of perifosine on AKT using a prostate cell line, PC-3, which is known to have a mutated, non-functional PTEN and thus to have constitutively activated AKT [8]. Their results demonstrated that very small doses of perifosine (5μM) blocked phosphorylation of AKT but did not decrease the total amount of AKT present in the cell. These effects occurred within 30 minutes of exposure to perifosine, suggesting this was an initial event rather than a secondary phenomenon due to perifosine effects elsewhere in the cell. When perifosine was removed, substantial recovery of AKT phosphorylation began within one hour, also suggesting that perifosine's effects are specific and not the result of general detergent effects. Perifosine also blocked the effects of insulin, EGF (epidermal growth factor), PDGF (platelet derived growth factor) and other ligands known to stimulate AKT activation. By contrast, perifosine did not affect growth factor-mediated PI3K activation. Finally, the investigators showed that perifosine blocked the localization of AKT to the cell membrane, consistent with the observation that PI3K activation was not affected but AKT phosphorylation was clearly diminished.

Investigators at the Netherlands Cancer Institute in Amsterdam obtained similar results using the epithelial cell line A431 and HeLa. [7] Three alkylphosphocholines, including both miltefosine and perifosine, were evaluated, and all appeared to inhibit AKT in a dose-dependent fashion. The alkylphosphocholines also blocked stimulation of AKT activity by insulin.

These effects on AKT were accomplished with perifosine levels that inhibit the growth of tumor cells *in vitro* and that can be achieved in the clinic. Preclinical studies undertaken mainly by Dr. Holland's lab at MSKCC have also demonstrated that perifosine is a potent inhibitor of AKT signaling in glia *in vitro*.

The activity of perifosine has been evaluated in numerous other human and murine cell lines. Cell lines demonstrating the greatest sensitivity to perifosine included KB (larynx), LNCaP (prostate), MAI-PaCa-2 (pancreas), DLD-1 (colon), and SK-HEP-1 (liver) (IC50 1.0 – 4.9 μg/mL) (Perifosine Investigator's Brochure 2008). In a soft agar tumor stem cell assay, the human KB (squamous) and murine L1210 (leukemia) cell lines were the most sensitive. In the methylene blue exclusion assay, the five most sensitive lines were: KB (squamous mouth), LU 65A (lung), LNCaP (prostate), PC-1 (lung) and Hep-2 (larynx) with IC50 values of 0.8 – 3.4 μg/mL. In the SRB/metabolic capacity assay, KM12 (colon), PC3 (prostate), M14 (melanoma), HOP-92 (lung) and SF295 (CNS) cancer cell lines were the most sensitive with IC50 values of 0.2 – 3.1 μg/mL. The majority of cell lines (in all assays) were more sensitive to perifosine than to miltefosine. Compared with leukemic cells, normal mouse bone marrow cells were shown to be relatively insensitive to perifosine *in vitro.*

Animal studies

The *in vivo* activity of perifosine has been evaluated via oral dosing in various schedules in several transplanted tumors, as well as in the dimethylbenzanthracene (DMBA) induced mammary tumor model of the rat. Two tumors resistant to conventional cytotoxics but sensitive to perifosine were the KB tumor (squamous cell) and AsPC-1 (human pancreatic carcinoma). *In vivo*, single oral doses of 511 mg/kg completely inhibited the growth of subcutaneously transplanted KB squamous cell tumors. When smaller doses (203 or 300 mg/kg) were administered twice at 7 day intervals, complete remissions lasting >63 days were achieved. Other xenografts in nude mice that responded well to perifosine included the Hep-2 laryngeal, R3327 rat prostate, SAS tongue, SC115 mouse breast and HCT-8 colon tumor. Perifosine was extremely active in the Sprague-Dawley rat model of dimethylbenzanthracene (DMBA) induced mammary tumors. Daily oral treatment using 21.5 mg/kg for 5 weeks led to complete regressions of tumors. Notably, complete regressions over the entire study period were also achieved by combining a high initial dose of 68.1 mg/kg followed by lower daily doses of 2.15 mg/kg over 20 days, whereas neither the loading dose nor low maintenance doses alone exhibited significant ant-tumor activity. Even large (4-8 grams), established DMBA induced mammary tumors responded to daily doses of 14.7 to 68.1 mg/kg over 28 days, with a persistent effect for more than 20 days after cessation of treatment.

Preclinical studies of perifosine given as a single oral dose (10 mg/kg) demonstrated near complete absorption, with an absolute bioavailability of 81% and 95% in male and female rats (Perifosine Investigator's Brochure, 2008). For doses ranging from 1 to 50 mg/kg, maximum plasma concentration (C_{max}) values ranged from 0.34 to 10.5 μg/mL. Time to achieve C_{max} was reached at a median of 16 to 32 hours following administration of perifosine. It should be noted however that while there was an approximately linear increase in C_{max} from 1 to 10 mg/kg, a 5-fold increase in dose from 10 to 50 mg/kg led only to a 3-fold increase in C_{max}. The volume of distribution (Vd) was twice the physical body volume, and the terminal half-life ($t_{1/2}$) was 120.5 – 171.4 hours.

A study of [^{14}C] excretion (urine, feces) in the rat after an oral dose of [^{14}C] perifosine at 10 and 50 mg/kg revealed the following proportions of renal and fecal excretion:

Table 1

Dose (mg/kg)	Excretion in Urine	Excretion in Feces
10	<24 hr 1.0%,<br>360 hr 20.4 %	<24 hr 24.6 %,<br>360 hr 42.9%
50	<24 hr 1.2%<br>48 hr 2.4%<br>72 hr 3.6%	<24 hr 22%,<br>48 hr 48.5%,<br>72 hr 52.6 %

In vitro binding of perifosine to human serum albumin or α1-acid glycoprotein ranged from 92 to 98% (Perifosine Investigator's Brochure, 2008). No concentration dependence of protein binding was observed, suggesting a high binding capacity of the plasma proteins for perifosine.

Activity in mouse gliomas

Perifosine has been shown to cross the blood/brain barrier in the mouse as the Holland lab also demonstrated that PRF also causes reductions in mouse glioma cell proliferation ($p<0.005$), as measured by MIB-1 (Ki67) labeling commonly used in human gliomas as shown in figure 2.[16]

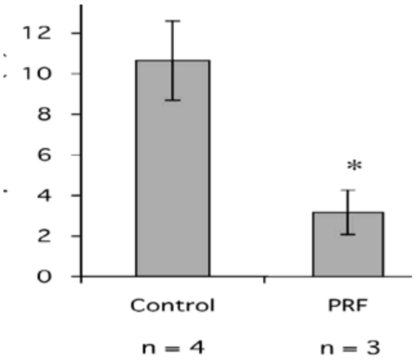

Fig 2: Ki-67^{+} cells (%) = MIB1

Preclinical Toxicity

In a subchronic toxicity study in rats, dose dependent toxicities were observed in the kidneys, gastrointestinal tract, skin, mammary glands, pituitary, hematopoietic tissue, spleen, ovaries, male genital tract, and eyes (Perifosine Investigator's Brochure, 2008). Effects on the hematopoietic tissue were characterized by an increase in cellularity for the bone marrow and an increased extramedullary hematopoiesis. Histopathologic changes seen in other tissues included evidence of chronic nephropathy, atrophy of hair follicles, atrophy of mammary glands, reduction of follicular development in the ovaries, degeneration of the germinal epithelium of the testes, atrophy/inactivation of prostate and seminal vesicles, secondary hypertrophy of the Leydig cells in the testes, and hypertrophy/hyperplasia of mucosal epithelium cells in stomach and small intestine. All toxicities, except those in the male genital tract and eyes, were reversible within 13 weeks. Most significant and not clearly reversible were ophthalmologic lesions, specifically, retinal degeneration and cataract formation. This toxicity was seen in rat but not in dog toxicity studies. Clinical chemistry revealed reversible increases in CK, BUN, SGOT, SGPT and reversible decrease of red blood cell parameters, total cholesterol, triglycerides, inorganic phosphorus and albumin and total protein.

In dogs, decreased food consumption, anorexia, diarrhea and vomiting were noted. Clinical chemistry revealed reduced glucose levels in the group receiving the highest dose. All changes were reversible during a 6-week recovery period. Histopathologically, minimal to mild degeneration of proximal tubular cells of the kidneys were found in male animals.

The no adverse effect level (NOAEL) was estimated to be < 2.15 mg/kg (12.9 mg/m^2) in rats and 2.15 mg/kg (43 mg/m^2) in dogs.

3.5 Perifosine Clinical Data

More than 1000 patients have been studied in Phase I and Phase II trials utilizing perifosine as a single agent in a variety of dose schedules. Detailed descriptions of each of these studies and the main results are included in the Investigational Brochure and the published references in Table 2. In addition, more than 750 patients have been studied utilizing perifosine in combination with various other drugs including gemcitabine, paclitaxel, docetaxel, doxorubicin, Doxil, capecitabine, pemetrexed, irinotecan, prednisone, dexamethasone, bortezomib, lenalidomide, imatinib mesylate, trastuzumab, aromatase inhibitors, sorafenib and sunitinib.

In the first Phase I trial a single dose of drug was administered weekly. The maximum tolerated dose was found to be 600 mg weekly [17]. Since both preclinical studies and early Phase I studies suggested that increases in plasma levels were not linear after a threshold (which might be about 400 mg), in some studies doses above 150 – 400mg were fractionated and administered every 4 - 6 hours to improve bioavailability and decrease C_{max}. It was found that higher single doses could be administered if the doses were divided. In one study by the National Cancer Institute doses of 900 mg, administered as two doses of 450 mg 6 hours apart, was considered the maximal tolerated dose [18]. In a Phase I trial at the University of Wisconsin a 900 mg loading dose was administered as 150 mg every 6 hours for 6 doses [19]. Finally, in a Phase I trial of weekly perifosine conducted by OCOG the maximum tolerated dose was determined to be 1200 mg when administered as 300 mg every 4 – 6 hours for 4 doses [20].

Daily doses were first evaluated with 21 day cycles followed by 7 days without drug [21]. It was concluded that the maximum tolerable dose was 200 mg per day. Subsequent Phase I and Phase II studies utilized doses of 100 mg continuously or 150 mg intermittently for 21 out of every 28 days following loading doses. (See Table 2) AOI studies #207 and 214 evaluated continuous doses of 50 and 100 mg without a loading dose, and in the case of trial #207 patients were randomized between a daily continuous dose and a weekly dose.

In general, tolerable doses identified in Phase I studies have been found to be less acceptable than expected in Phase II trials.

MSKCC experience

Protocol 06-044 is a phase II study that involves perifosine monotherapy for adults with recurrent/progressive malignant gliomas. Approximately 20 patients were enrolled at this time and treated with a 600 mg loading dose on day 1 (150 mg x 4) followed by 100 mg nightly

thereafter. This dose was well tolerated (Society for Neuro-Oncology, 2007). Accrual to that study continues.

Dosing schedules from other studies are shown in Table 2 below.

Table 2 Phase I and Phase II trials of perifosine used as a single agent.

Sponsor Trial #	Locale	Phase	Schedule	Doses in mg	Number of Patients	Reference
Zentaris #3040	Germany	I	Weekly Single Bolus	200 - 800	36	
Zentaris #3087	Germany	I	Weekly Bolus Enteric Coated	200 – 450	13	
Keryx #201A	US OCOG	I	Weekly Divided Doses	900 – 1800	20	[19]
NCI T98-0065	US Bethesda	I	Loading Divided + Intermittent Daily	300 – 1500 50 – 250	31	
NCI T98-0036	US Wisconsin	I	Loading Divided + Continuous Daily	400 – 900 50 – 150	42	[20]
Zentaris #3079	Netherlands	I	Intermittent Daily	50 – 350	22	[21]
NCI #5938	US Chicago	II Head/Neck	Loading Divided + Continuous Daily	900 100	19	[22]
NCI #5970	US Bethesda	II Prostate	Loading Divided + Intermittent Daily	900-300 150	19	[23]
NCI #5972	US Madison	II Sarcoma	Loading Divided + Continuous Daily	600 100	23	[24]
NCI 5974	Canada Vancouver	II Sarcoma	Loading Divided + Intermittent Daily	900 150	16	[25]
NCI #5975	Canada Toronto	II Melanoma	Loading Divided + Intermittent Daily	900-300 150	18	[26]
NCI #5978	US Davis, CA	II Prostate	Loading Divided + Continuous Daily	900 100	25	
NCI #5982	Canada Toronto	II Breast	Loading Divided + Intermittent Daily	900 150	18	[27]
NCI #5983	Canada Toronto	II Pancreas	Loading Divided + Intermittent Daily	900-300 150	19	[28]
NCI E1202	US ECOG	II Pancreas	Loading Divided + Continuous Daily	900 100	10	[29]
AOI #201B*†	US OCOG	II Lung	Weekly Divided Continuous Daily	900 150	68+	
AOI #207*†	US OCOG	II “All comers”	Weekly Divided Continuous Daily	900-1200 50 – 100	489	
AOI #209	US OCOG	II Sarcomas	Weekly Divided	900-1500	91	
AOI #212	US Multicenter	II Multiple Myeloma	Continuous Daily	150	67	
AOI #214*	US SARC	II Sarcoma	Continuous Daily	100	57	
AOI #217*	US OCOG	II Acute Leukemia	Continuous Daily	150	19	
AOI #221*	US DFCI	II Waldenström’s	Continuous Daily	150	32	
AOI #222	US MSKCC	II Glioma	Loading Divided + Continuous Daily	600 100	24	

AOI #228**	US Multicenter	II Renal Cell	Continuous Daily	100	8	
AOI #231**	US OCOG	II Renal Cell	Continuous Daily	100	0	

*Trial still open and accruing   ** Recently opened and accruing
† Patients randomized to different doses and schedules of perifosine

Toxicity

In all Phase I and Phase II trials, dose-limiting toxicity has been gastrointestinal and fatigue. In summary of all these trials: there was approximately 30% grade 1-2 nausea but only 2% grade 3-4 nausea, 20% grade 1-2 vomiting but only 2% grade 3-4 vomiting, 25% grade 1-2 diarrhea but only 4% grade 3-4 diarrhea, and 15% grade 1-2 fatigue but only 4% grade 3-4 fatigue. Gastrointestinal side effects and fatigue are both more common than any other forms of toxicity but are also clearly dose and schedule related (Table 4).

Other side effects occur much less frequently. Those observed in the first 758 patients are shown in Table 3. Most of the adverse events occur at a low grade. Other than gastrointestinal toxicity and fatigue, the incidence of grade 2-4 toxicities, rarely exceed 10%. Anemia and lymphopenia are the most common in this category (other hematological abnormalities were extremely rare). Hyperglycemia has been observed in 20% of patients, but this was grade 1 hyperglycemia in most cases (Table 3).

Table 3 shows adverse events exclusive of common gastrointestinal events and fatigue, that occurred with a frequency of $\geq$ 2% reported by 1047 patients in Phase I/II trials of perifosine that utilized a variety of doses and schedules. Events that were reported but thought unlikely to be related to perifosine are not included in this list.

Table 3 Adverse events, exclusive of common gastrointestinal events and fatigue, that occurred with a frequency of > 2% reported by 1047 patients in phase I/II trials of perifosine that utilized a variety of doses and schedules. Events that were reported but thought unlikely to be related to perifosine are not included in this list.

	ADVERSE EVENT	Grade (%) 1	2	3	4
**CONSTITUTIONAL SYMPTOMS**	Weight loss	4	2	<1	
	Fever	3	<1	<1	<1
	Insomnia	2	1		
	Sweating	2	1		
**PAIN**	Abdominal pain	4	3	1	
	Headache	3	1	<1	
**MUSCULOSKELETAL**	Joint pain	2	2	1	
	Pain in extremity	1	1	1	
	Muscle weakness	1	2	<1	
**DERMATOLOGY/SKIN**	Rash	3	<1	<1	
**LYMPHATICS**	Edema limbs	1	1	<1	
**NEUROLOGY**	Dizziness	2	1	<1	
	Peripheral Sensory Neuropathy	2	1	<1	
**UNCOMMON GI**	Anorexia	11		2	
	Constipation	6	2	<1	<1
	Flatulence	4	2		
	Dyspepsia	3	1		
	Dehydration	1	3	2	
	Mucositis Oral	2	1		
	Taste alteration	3	1		
**PULMONARY**	Cough	2	<1	<1	<1
	Dyspnea	1	1	1	
	Hiccough	2	1		
**LABORATORY HEMATOLOGIC**	Hemoglobin decreased	15	11	2	<1
	Lymphopenia		11	6	2
	Platelet count decreased	4	1	1	1
	Leukopenia	3	2	1	<1
	Neutrophil count decreased		1	1	1
**LABORATORY NON-HEMATOLOGY**	Alanine aminotransferase increased	7	2	1	
	Aspartate aminotransferase increased	5	3	1	
	BUN increased	13			
	Creatinine increased	7	3	1	<1
	Gamma-Glutamyl Transferase Increased	2	1	<1	
	Hyperglycemia	13	7	2	<1
	Hypoalbuminemia	11	8	2	<1
	Hyponatremia	9		2	1
	Proteinuria	2	2	<1	1
	LDH Increased	13			
	Hyperbilirubinemia	1	1		1
	Alkaline phosphatase increased	5	2	1	
	Hypocalcemia	5	3	<1	<1
	Hyperkalemia	2	1	1	1
	Hypercalcemia	2	1	<1	<1
	Hypernatremia	2	<1		<1
	Hyperuricemia	3		<1	1
	Hypoglycemia	2	1		1
	Hypokalemia	4		1	<1
	Hypophosphatemia	0	2	1	<1

Table 4. Gastrointestinal toxicity observed with various dose-schedules of perifosine used in the NCI Phase II trials and in AOI trials #201, 207, 209 and 214.

Schedule		Daily With A Loading Dose		Weekly In Divided Doses			Daily Continuous No Loading Dose		
		Intermittent	Continuous						
Dose		900/150**	900/100**	900	1200	1500	50	100	150
Number of Patients		89	65	169	125	70	142	120	41
Adverse Event		%	%	%	%	%	%	%	%
Nausea	Grade 0	26	52	30	30	20	56	51	34
	Grade 1	30	34	50	30	30	31	33	39
	Grade 2	35	12	19	34	41	8	12	24
	Grade 3	9	2	1	6	7	5	4	2
	Grade 4	0	0	0	0	1	1	0	0
Vomiting	Grade 0	35	72	43	50	29	69	71	56
	Grade 1	36	17	39	26	37	16	16	31
	Grade 2	25	9	17	20	29	9	12	10
	Grade 3	4	2	1	4	4	5	2	3
	Grade 4	0	0	0	0	1	1	0	0
Diarrhea	Grade 0	29	51	29	25	13	61	65	46
	Grade 1	29	38	53	33	40	29	28	41
	Grade 2	27	9	10	26	30	9	7	10
	Grade 3	11	2	8	16	16	1	0	2
	Grade 4	0	0	0	0	1	0	0	0
Fatigue	Grade 0	-	-	62	58	60	64	65	42
	Grade 1	-	-	20	11	20	14	18	17
	Grade 2	-	-	15	17	13	15	13	27
	Grade 3	-	-	4	11	7	6	4	15
	Grade 4	-	-	0	2	0	1	0	0

**On the intermittent schedule, loading dose on day 1, cycle 1 was 900 and on subsequent cycles was 300 on day 1; daily doses given on days 2–21 of 28 day cycle. On the continuous schedule, loading dose on day 1 was either 600 or 900 mg, depending on investigator, and there were no cycles.

Pharmacokinetic Data

The half-life of perifosine is about 100 hours. When a daily dose is administered without a loading dose, steady state levels are reached in 8 – 14 days. Therefore, to achieve rapid steady state serum levels, we do plan a loading dose followed by maintenance dose in this trial.

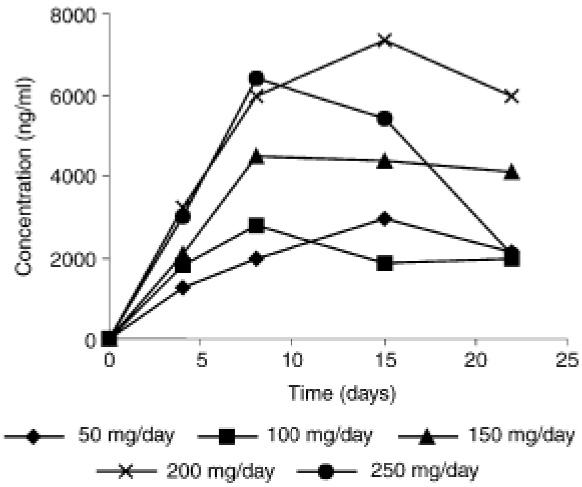

Figure 3 Trough plasma concentrations of perifosine in patients treated in trial #3079 with daily doses of perifosine for 21 out each 28 day cycle. Doses between patient cohorts were increased from 50 mg per day to the maximally tolerate dose of 250 mg per day. There were either 3 to 5 observations at all of the time points in the chart with the exception of the day 8 value for 100 mg and the day 22 value for 250 mg. For each of these there were only 2 measurements [21].

The absolute values shown here may not correlate well with tumor levels of perifosine since it has been shown in preclinical models that the intratumoral levels may be as much as 1 log higher than plasma levels [30].

In studies using a radiolabeled perifosine in tumor bearing mice, maximum plasma concentrations of 5.7 µg/ml were reached 22 hours after a single dose of 40 mg/kg [30]. In these mice the $t_{1/2}$ was 137 $\pm$ 20 hours. Maximum intratumoral levels of 60 to 100 µg/g, which varied with the tumor type, were reached 48 hours after the single dose. This represents more than a 1 log difference between plasma and intratumoral levels. These levels were then maintained for up to 144 hours. There was very little metabolism of drug, which contrasted with first generation alkylphospholipid, miltefosine. Small intestine concentrations of perifosine were higher than the concentration in other organs.

Efficacy observed in Phase II trials

A recent review (unpublished) of results from Phase I and Phase II studies demonstrated that perifosine induced 21 partial responses in various malignancies including renal cell and hepatocellular carcinoma, soft tissue sarcoma, GIST tumors, mesothelioma, carcinoma of the

appendix, and multiple myeloma. Disease stabilization, defined as time on treatment without progression for at least 6 months was seen in 20 tumor types, including melanoma, carcinoid, prostate, head and neck, breast, and small cell lung cancer.

There is no evidence of a dose-response to perifosine, but the overall database and individual randomized trials are still too small to rule out a modest improvement in outcomes related to dose. Responses have been seen at both the smallest, least toxic dose, 50 mg daily, as well as with higher weekly doses. The most compelling data supporting roughly equivalent efficacy from lower and higher doses is from an analysis of all accumulated sarcoma data, which were presented at ASCO in 2007 [31], and from the an analysis of outcomes for renal cancer patients in the randomized Phase II trial, AOI #207.

One hundred forty-five patients with soft tissue sarcoma have been enrolled in either one of three Phase I or four Phase II trials. Eighty-five of these patients were from AOI Trial #209 which was conducted by OCOG. The patients from these studies were retrospectively divided into two cohorts defined by the dose of perifosine that the patient received as shown in Table 5. The higher doses were associated with considerably more toxicity, and this was the basis for discontinuing treatment without evidence of progression for 32% of the patients receiving the higher doses compared to only 12% of those on lower doses. Patients were considered fully evaluable for response if they had received at least 2 months (i.e. 2 cycles) of treatment and had documentation of response or progression before being taken off study. The RECIST criteria were used to define response, but patients had to remain on treatment without progression for at least 4 months to be scored as having stable disease. The definition of clinical benefit rate, either a partial response or disease stabilization for at least 4 months, had previously been used to assess benefit from an mTOR inhibitor [32]. The overall clinical benefit rate was 52% using only evaluable patients and 30% using all patients enrolled as a denominator. When only fully evaluable patients were considered, there was no significant difference in clinical benefit rate. The responders were all treated with one of the lower doses. The histology of the four tumors with a partial response was chondrosarcoma, extra-skeletal myxoid chondrosarcoma, leiomyosarcoma, and desmoids tumor. The patient with the leiomyosarcoma had a particularly durable response. This patient had multiple sites of metastases including lung, bone, scalp and retroperitoneum, received 50 mg of perifosine daily for almost 17 months, had no gastrointestinal toxicity or fatigue, and remained in remission for more than 3 years. As with some other patients who have responded to perifosine, the onset of response was slow and could not be scored as a partial response until after 4 months of treatment.

Table 5 Perifosine Efficacy in Soft Tissue Sarcoma .

	**Number of Patients**		
	**All Doses**	**Lower Doses**<br>**Weekly ≤ 900 mg**<br>**Loading dose ≤ 900 mg**<br>**Daily dose ≤ 100 mg**	**Higher Doses**<br>**Weekly 1200 mg or 1500 mg**<br>**Loading dose ≥ 1200 mg**<br>**Daily dose ≥ 150 mg**
**Total enrolled**	145	76	69

IRB PB

Amended: 8/13/14

**Removed due to toxicity, no evidence of progression**	-31	-9	-22
**Removed for progression in < 2 months**	-32	-15	-17
**Fully evaluable for CBR***	82	52	30
**Partial response**	4	4	0
**Stable ≥ 4 months**	39	23	16
**Clinical benefit rate based on evaluable**	52%	52%	53%
**Clinical benefit rate based on all patients**	30%	36%	23%

At the 2007 Society for Neuro-Oncology meeting, preliminary results of MSKCC trial 06-044 (phase II trial of perifosine monotherapy for adults with recurrent/progressive malignant glioma) were reported. There were responses in patients with anaplastic gliomas, and accrual for such patients was therefore recently expanded and continues. However, there was no evidence of activity in patients with recurrent GBM; therefore, accrual for patients with recurrent GBM was stopped as mandated by an early stopping rule in the trial.

Recently, at the 49th Annual Meeting of American Society of Hematology, initial results of a multicenter phase 1/2 trial of perifosine with bortezomib were presented in relapsed or relapsed/refractory multiple myeloma patients previously treated with bortezomib. Eighteen patients (median age 64 yrs) with advanced MM (83% relapsed and refractory) were enrolled in one of four cohorts. Patients had a median of 5 lines of prior therapy and 100% of patients were previously treated with at least one course of therapy on bortezomib. Perifosine was escalated from 50 to 100 mg qd while bortezomib was escalated from 1.0 to 1.3 mg/mm2. No dose-limiting toxicity and no grade 3 peripheral neuropathy were reported. Toxicities were generally well managed and tolerated. Dexamethasone 20mg (day of and day after each Velcade dose) was added in patients with progressive disease (PD) on perifosine plus Velcade alone. Sixteen patients on either Velcade plus perifosine alone or with dexamethasone were evaluable for response, assessed by modified EBMT/Blade criteria, with results as follows: combination of perifosine and bortezomib (+/- dexamethasone) was well-tolerated and is active in heavily pre-treated and relapsed/refractory multiple myeloma, including bortezomib- resistant patients. An overall response rate of 56% (CR + PR + MR) was reported with an additional 31% of patients achieving stable disease (SD). The phase 2 portion of the study is currently ongoing. In addition, in a phase 2 of perifosine +/- dexamethasone also in the treatment of pretreated multiple myeloma, perifosine has demonstrated modest activity whereby 66% of patients (33/50) achieving stable disease. In addition, the combination of dexamethasone and perifosine (dexamethasone was added if progression on perifosine alone) resulted in an overall response rate of 35% (10/29; CR+PR+MR) with an additional 52% (15/29) of patients achieving stable disease. Thus, perifosine appears to be a promising agent for combination therapies.

3.6 Potential impact on metabolism by hepatic enzyme inducing antiepileptic drugs (EIAEDs)

There is increasing evidence that brain tumor patients receiving enzyme-inducing anti-epileptic drugs (EIAEDs) have markedly altered pharmacokinetics, resulting in accelerated drug metabolism. This may result in decreased levels of certain chemotherapeutic agents when administered at conventional doses. Failure to achieve adequate plasma levels of such agents may account for the lack of efficacy in past brain tumor trials.

Perifosine is a potential substrate for the cytochrome P450 system, and the escalating concentrations of perifosine were tested for inhibition of the liver microsomal cytochrome P450 system in vitro (table 6). Enzyme activities associated with CYPlA2 (7-ethoxyresorufin 0-deethylation), CYP2A6 (coumarin 7-hydroxylation), CYP2B6 (S-mephenytoin N-demethylation), CYP2C9 (tolbutamide methyl-hydroxylation), CYP2C19 (S-mephenytoin 4'-hydroxylation), CYP2D6 (dextromethorphan 0-demethylation), CYP2E1 (chlorzoxazone 6-hydroxylation) and CYP3A415 (testosterone 6P-hydroxylation) were determined using enzyme-specific probe compounds incubated with pooled human liver microsomal samples.

In the presence of perifosine, a moderate inhibitory effect was observed on CYP2A6 activity (50%), and a slight inhibitory effect on CYP3A4 activity (29%), in both cases only at the highest concentration (100 µM). No inhibitory effect was observed on CYP2C19 activity by perifosine at any of the concentrations tested. Enzyme activities associated with CYPIA2 (7-ethoxyresorufin 0-deethylation), CYP2B6 (S-mephenytoin Ndemethylation), CYP2C9 (tolbutamide methyl-hydroxylation), CYP2D6 (dextromethorphan 0-demethylation), and CYPZEI (chlorzoxazone 6-hydroxylation) were not inhibited by perifosine at any of the concentrations (0.25 - 100 µM).

Table 6: The Effect of Perifosine on CYP1A2, CYP2A6, CYP2B6, CYP2C9, CYP2C19, CYP2D6, CYP2E1 AND CYP3SA4/5 Activities in Pooled Human Liver Microsomes

Perifosine	Percentage of Activity Remaining							
Conc (µM)	CYP1A2	CYP2A6	CYP2B6	CYP2C9	CYP2C19	CYP2D6	CYP2E1	CYP3A4/5
0.0	100.0	100.0	100.0	100.0	100.0	100.0	100.0	100.0
0.25	96.4	97.7	100.1	101.3	98.4	96.0	99.8	102.2
1	101.2	101.2	100.7	96.6	96.8	94.1	100.4	101.3
5	100.8	90.8	100.1	95.7	102.0	98.5	95.8	103.0
10	98.8	94.7	98.0	95.3	97.6	95.9	95.0	97.0
25	99.9	88.9	100.8	96.0	103.1	98.5	96.5	94.5
50	97.8	88.7	101.0	95.8	113.9	94.3	89.1	98.4
100	113.6	46.1	99.9	98.0	106.4	88.1	86.0	71.3

3.7 Phase I experience in children

Lack of toxicity – specifically no ≥ grade 3 and no dose-limiting toxicities - in this 08-091 study eventuated in dose escalation beyond dose level #3 which was the highest planned dose level when the protocol was devised and initiated. Of note in this regard, the absence of hematologic, hepatic, and unexpected toxicities prompted single patient usage of Perifosine at dose level #3 in two patients who did not qualify for the formal protocol because they each had grade 3-4 abnormalities of liver function enzymes – and both patients tolerated the Perifosine treatment well, without any ≥ grade 2 toxicity.

Preliminary PK studies revealed steady state serum levels of 14.1±4 µM at dose level #1, 32.8±8.1 µM at dose level #2, and 31.6±7.8 µM at dose level #3. These results suggest that dose level #3 will prove to be optimal.

As regards anti-tumor activity, notable findings include 12 months (then change to other therapy) and 18+ months of progression-free survival of two patients with metastatic chemoresistant neuroblastoma.

4.0 OVERVIEW OF STUDY DESIGN/INTERVENTION

4.1 Design

This is a single arm, phase I safety study in which eligible patients will receive a loading dose of oral perifosine on the first day, followed by a maintenance dose starting on the second day until progression as described in section 9.1 where each patient is assigned to a group according to their body surface area (BSA).

4.2 Intervention

Following a diagnosis of tumor recurrence or progression, all patients will receive perifosine monotherapy until toxicity, progression, or death. Imaging (MRI/CT) will be performed at baseline and every 2 months. Serum markers and/or tissue sampling will also be assessed every 2 months if used to follow therapeutic response.

5.0 THERAPEUTIC/DIAGNOSTIC AGENTS

Perifosine (NSC # 639966; IND # 69,383)

Chemical Name: 1,1-dimethyl-4-[[(octadecyloxy)hydroxyphosphinyl]oxy]-piperidinium inner salt

Other Names: Octadecylphosphopiperidine (OPP), D-21266, KRX-0401

Molecular Formula: $C_{25}H_{52}NO_4P$

M.W.: 461.66 g/mol

CAS No.: 157716-52-4

How Supplied: Perifosine is supplied as a film-coated tablet containing 50 mg of active ingredient.

Storage: Store closed containers at room temperature (20-25 ° C). Perifosine is hygroscopic, so containers must be kept closed and dry. Perifosine tablets must be dispensed in the original container.

Stability: Stability testing program ongoing. Perifosine has a demonstrated shelf stability of at least 3 years.

Route of Administration: Oral with meals (tablets must be taken whole)

Potential Drug Interactions: None reported.

Availability: Perifosine is an investigational agent supplied to investigators by Aeterna Zentaris GmbH.

Agent Accountability: The Investigator, or a responsible party designated by the Investigator, must maintain a careful record of the inventory and disposition of all agents received from Aeterna Zentaris GmbH using a Drug Accountability Record Form.

6.0 CRITERIA FOR SUBJECT ELIGIBILITY

6.1 Subject Inclusion Criteria

- Any solid tumor that has failed standard therapy
- Patient must have evidence of tumor by CT, MRI, MIBG scan, serum markers, or tissue sampling.
- Age ≤ 21 years,
- Karnofsky/Lansky performance status ≥ 50% (Karnofsky score for age > 16 years and Lansky score for age ≤ 16 years)

- ANC ≥ 1000 at least 24 hours off GCSF
- Platelets ≥ 75k at least one week off platelet transfusions
- Hg ≥ 8g/dL at least one week off PRBC transfusion
- AST ≤ 3 x the upper limit of normal
- ALT ≤ 3 x the upper limit of normal
- Total bilirubin ≤ 2.0 mg/dl
- serum creatinine ≤ 1.5 x the upper limit of normal for age, or calculated creatinine clearance or nuclear GFR ≥ 70 ml/min/1.73 m^2.
- ≥ 3 weeks since last non-nitrosourea chemotherapy
- ≥ 6 weeks since last nitrosoureas
- ≥ 4 weeks since last RT
- Patients must agree to practice adequate contraception. Females of childbearing potential must have a negative serum B-HCG pregnancy test documented within 14 days prior to drug initiation. Females must not be breast feeding.
- Patients must be able to swallow tablets whole

6.2 Subject Exclusion Criteria

- Pregnancy
- Patients must not have active infection or serious intercurrent medical illness.
- HIV-Positive patients receiving combination anti-retroviral therapy are excluded from the study due to possible retro-viral drug interactions. HIV testing not required.
- Patients must not be taking EIAEDs (EIAEDs listed in section 9.4.7). If patients were previously on EIAEDs that have been discontinued, patients must have been off the agent for at least 2 weeks prior to registration.

7.0 RECRUITMENT PLAN

Patients will be offered the opportunity to participate in this trial if they meet the eligibility criteria. There will be no discrimination against females or minorities. Informed consent will be obtained from the patient, or if they are non-emancipated minors, their parent or legal guardian. Consent will be obtained by an investigator authorized to obtain consent. Patients will not receive any payment for their participation in this study.

8.0 PRETREATMENT EVALUATION

8.1 Pretreatment Evaluation for perifosine monotherapy

THE FOLLOWING ARE REQUIRED WITHIN TWO WEEKS (with the exception of the MIBG scan) PRIOR TO STUDY ENROLLMENT:

- Complete history and physical exam and KPS/LS

- Prior to beginning perifosine, all patients should be questioned about a history of hyperuricemia and/or gout. Patients with a known history of hyperuricemia and/or gout should receive prophylactic treatment with allopurinol. If severe gout does occur or uric acid levels increase, the dose of allopurinol should be increased at physician discretion.
- Height and weight.
- CBC including WBC differential and platelet count.
- Comprehensive chemistry panel.
- Uric Acid
- LDH
- Phosphorus
- Magnesium
- βHCG for women of childbearing age (must be done within 14 days of Perifosine initiation; some patients may require a repeat test if start of treatment occurs more than 14 days after the screening test).
- Contrast enhanced MRI (with MR Perfusion if possible) or CT. Patients unable to undergo an MRI will undergo contrast enhanced CT scans.
- MIBG scan or PET scan for patients with neuroblastoma is required within 3 weeks of study enrollment.

9.0 TREATMENT/INTERVENTION PLAN

Dosing schedule will be as described in section 9.1. For the purpose of this trial a cycle will be defined as 28 days. Perifosine tablets should be taken with food. For patients enrolled after March 16, 2011, a maximum of a 4 week supply of Perifosine may be prescribed/dispensed by the MSKCC pharmacy.

9.1 Dosing- Perifosine monotherapy

We will be testing five dose levels. In addition, one dose will be available for deescalation, in the situation that the 1st dose level proves to be the level at which DLT occurs. As perifosine is only available in 50mg tablets, dosing will not be per surface area but in five groups where the grouping is based on BSA. In addition, given the long half-life of the drug, the drug does not have to be dosed daily. Perifosine will be given as an oral load dose on day 1. On day 2, patients will start the oral maintenance dose.
At the end of each cycle, the patient's BSA will be re-calculated. If the BSA is different from baseline then the oral maintenance dose will be modified to accommodate the newly calculated BSA.

Dose level # -1

BSA (0.40-0.59) - Load of 50mg (oral) on D#1 and maintenance dose of 50mg (oral) once a week starting on D#2.

BSA (0.60-0.79) - Load of 50mg (oral) on D#1 and maintenance dose of 50mg

(oral) every five days starting on D#2.
BSA (0.8-1.20) - Load of 100mg (oral) on D#1 and maintenance dose of 50mg (oral) twice a week starting on D#2.
BSA (1.21-1.6) - Load of 150mg (oral) on D#1 and maintenance dose of 50mg (oral) three times per week starting on D#2.
BSA >1.6 - Load of 150mg (oral) on D#1 and maintenance dose of 50mg (oral) four times per week starting on D#2.

Dose level # 1 (starting dose level)
BSA (0.4-0.59)- Load of 50mg (oral) on D#1 and maintenance dose of 50mg (oral) every four days starting on D#2.
BSA (0.6-0.79) - Load of 50mg (oral) on D#1 and maintenance dose of 50mg (oral) every three days starting on D#2
BSA (0.8-1.20) - Load of 100mg (oral) on D#1 and maintenance dose of 50mg (oral) every other day starting on D#2.
BSA (1.21-1.6) - Load of 150mg (oral) on D#1 and maintenance dose of 50mg (oral) five days per week starting on D#2.
BSA >1.6 - Load of 150mg (oral) on D#1 and maintenance dose of 50mg (oral) daily starting on D#2.

Dose level # 2
BSA (0.4-0.59) – Load of 100mg (oral) on D#1 and maintenance dose of 50mg (oral) every other day starting on D#2.
BSA (0.6-0.79) - Load of 100mg (oral) on D#1 and maintenance dose of 50 mg (oral) daily five days per week starting on D#2.
BSA (0.8-1.20) - Load of 100mg BID (oral) on D#1 and maintenance dose of 50mg (oral) daily starting on D#2
BSA (1.21-1.6) - Load of 150mg BID (oral) on D#1 and maintenance dose of 100mg (oral) daily five days per week starting on D#2.
BSA >1.6 - Load of 150mg BID (oral) on D#1 and maintenance dose of 100mg (oral) daily starting on D#2.

Dose level# 3
BSA (0.4-0.59) - Load of 100mg (oral) on D#1 and maintenance dose of 50mg (oral) daily five days per week starting on D#2.
BSA (0.6-0.79) - Load of 100mg (oral) on D#1 and maintenance dose of 50mg (oral) daily starting on D#2.
BSA (0.8-1.20) - Load of 100mg BID (oral) on D#1 and maintenance dose of 50mg

(oral) daily alternating with 100mg daily starting on D#2.
BSA (1.21-1.6)-Load of 150mg BID (oral) on D#1 and maintenance dose of 100mg (oral) daily starting on D#2.
BSA >1.6 -Load of 150mg BID (oral) on D#1 and maintenance dose of 100mg (oral) daily alternating with 150mg (oral) daily starting on D#2.

Dose level#4
BSA (0.4-0.59) - Load of 100mg (oral) on D#1 and maintenance dose of 50mg (oral) daily starting on D#2.
BSA (0.6-0.79) - Load of 100mg (oral) on D#1 and maintenance dose of 50mg (oral) daily alternating with 100mg (oral) daily starting on D#2.
BSA (0.8-1.20) - Load of 100mg BID (oral) on D#1 and maintenance dose of 100mg (oral) daily starting on D#2.
BSA (1.21-1.6)-Load of 150mg BID (oral) on D#1 and maintenance dose of 150mg (oral) daily six days per week and 100mg(oral) daily for one day per week starting on D#2.
BSA >1.6 -Load of 150mg BID (oral) on D#1 and maintenance dose of 150mg (oral) daily alternating with 200mg (oral) daily starting on D#2.

Dose level# 5
BSA (0.4-0.59) - Load of 100mg (oral) on D#1 and maintenance dose of 50mg (oral) daily **five** day per week and 100mg daily **two** days per week starting on D#2.
BSA (0.6-0.79) - Load of 100mg (oral) on D#1 and maintenance dose of 100mg (oral) daily **five** days per week and 50mg daily **two** days per week starting on D#2.
BSA (0.8-1.20) - Load of 100mg BID (oral) on D#1 and maintenance dose of 100mg (oral) daily alternating with 150mg (oral) daily starting on D#2.
BSA (1.21-1.6)-Load of 150mg BID (oral) on D#1 and maintenance dose of 200mg (oral) daily alternating with 150mg(oral) daily per week starting on D#2.
BSA >1.6 -Load of 150mg BID (oral) on D#1 and maintenance dose of 200mg (oral) daily starting on D#2.

If all of the above dose levels are well- tolerated and no DLTs are encountered, then additional dose levels may be added via an amendment to the protocol.

9.1.1 Dose Escalation - Deescalation

Patients will be treated in cohorts of size three to six, starting at dose level 1. The dosage will be escalated if the clinical toxicity is declared acceptable (below). One dose is available for deescalation, if the 1st dose level proves to be the level at which DLT occurs.

Each patient is evaluated for toxicity after being treated at a particular dose level for at least 4 weeks after the first dose of perifosine (one cycle). Regardless of how long the perifosine continues to be administrated, the toxicity evaluation will be based on the first cycle of therapy. No intrapatient dose escalation will be performed. DLT is defined in section 9.3.

The dose escalation-deescalation scheme is as follows:

- If zero (0) of 3 subjects at a dose level experience a DLT after one cycle of perifosine, then dose escalation will occur and 3 subjects will be treated at the next higher dose level.
- If 1of 3 subjects at a dose level experiences a DLT after one cycle of perifosine, then 3 additional subjects will be treated at the same dose level. Escalation at the next dose level will proceed only if none of the additional subjects experienced a DLT.
- If ≥ 2 of 3 subjects at a dose level or ≥ 2 of 6 subjects at an expanded dose level experience a DLT after one cycle of perifosine, dose escalation will stop and the prior dose level will be considered the MTD. If this occurs at dose level 1, then the next three patients will be enrolled at dose level -1.
- If only three patients were treated at a dose under consideration as MTD, an additional three patients will be treated at that level to confirm previous results.

9.2 Pharmacokinetics (all patients)

In addition to baseline serum, all patients will have weekly serum drawn at baseline and during weeks 2-4 of cycle 1.

Jill M. Kolesar, Pharm. D. at the University of Wisconsin will conduct PK studies. Aeterna Zentaris GmbH subcontracted Dr. Kolesar to perform such assays.

9.2.1 Handling and Shipping of Serum for Perifosine Measurement:

A. Serum

At each time point, heparinized blood is collected into a plastic vacutainer to minimize adhesion of perifosine. Plasma is separated by centrifugation and stored in polypropylene cryovials at -70°C until assayed. Perifosine in plasma is measured by a validated reversed phase liquid chromatography/electrospray mass spectrometry method by Jill M. Kolesar, Pharm.D at the University of Wisconsin, Comprehensive Cancer Center who has been subcontracted by Aeterna Zentaris GmbH to perform these analyses. An LC/MS analytical method used in NCI sponsored phase I trials (Woo EW, Messmann R, Sausville EA, Figg WD. Quantitative determination of perifosine, a novel alkylphosphocholine anticancer agent, in human plasma by reversed-phase liquid chromatography-electrospray mass spectrometry. J Chromatogr B Biomed Sci Appl. 2001 Aug 15;759(2):247-57), is currently available for plasma samples at the Univ. of Wisconsin, and

will be used for this study. The pharmacokinetic characteristics of perifosine in patients will be evaluated using WinNonlin modeling software.

Sample processing and handling outline:

1) A pharmacokinetic sample will be obtained from each patient, at the times outlined. One sodium heparin 10cc green top with >7ml of blood will be obtained and shipped to Jill M. Kolesar, Pharm.D at the University of Wisconsin for further analysis.

2) Pharmacokinetic samples will be obtained in association with perifosine administration at pretreatment, and weekly during weeks 2-4 (at least 5 days apart) of cycle 1. In addition, an additional pharmacokinetic sample will be obtained if a patient were to encounter a DLT.

3) Peripheral blood is to be drawn into heparinized vacutainer (green top) provided in the sample collection kit.

4) As soon as possible, centrifuge the blood samples at 900g for 15 minutes (or use the local laboratory Standard Operating Procedure) to separate plasma.

5) For each sample collected, pipet duplicate aliquots of plasma, 2 ml each, in polypropylene cryovials (Nunc) provided in the sample collection kit. Aliquot 1 is the sample and aliquot 2 is the duplicate. The patient's initials, study number, time and date the sample was obtained should be recorded on each cryovial.

6) Place the appropriate labels, included in the sample collection kit, onto the tubes and freeze at -70°C until shipped.

7) Sample Shipping:

- Samples are to be shipped overnight in sufficient dry ice to keep samples frozen using the airbill and shipping box provided in the kit.

- Please place all 4 nunc sample tubes into the plastic bag provided. Do not ship samples and duplicates in the same box. Save the duplicates and ship them with your next patient.
  - Sample shipments can be batched.

- Place the patient's PK research assessment sheet in a separate plastic bag and then place it in the shipping box.

- Please contact the central lab before shipping samples
  Amy Dresen (608) 263-5369 or Jill Kolesar (608) 262-5549
  or

Email Marcy Pomplun: mpomplun@wisc.edu

- The laboratory is open to receive samples Monday through Friday. NOTE: **DO NOT ship samples on Fridays or on days prior to holidays.**

- Ship to: **Jill M. Kolesar, Pharm.D**
  Analytical Instrumentation Laboratory for Pharmacokinetics, Pharmacodynamics and Pharmacogenetics (3P)
  University of Wisconsin
  Comprehensive Cancer Center Room K6/570
  600 Highland Avenue
  Madison, WI 53792-5669

9.3 Definition of Dose-Limiting Toxicity (DLT) and treatment modifications

Toxicities will be graded according to the CTCAE v 3.0.[33]

For grade 1 perifosine possibly, probably, or definitely related adverse events, treatment with perifosine will not be interrupted.

For grade 2 perifosine possibly, probably, or definitely related adverse events:

- For nausea, vomiting and diarrhea, maintain dosing with symptomatic treatment. The dose of perifosine may be repeated if emesis occurs within 30 minutes of taking the tablet(s) OR all the tablets are seen in the emesis.
- For persistent nausea, vomiting or diarrhea, despite symptomatic treatment, patient may continue with home intravenous hydration.

For grade 3 or 4 perifosine possibly, probably, or definitely related adverse events

DLT is defined as:

- Any non-hematological toxicity grade ≥3 (except for grade 3 nausea, vomiting, and diarrhea that can be controlled within 24 hours with supportive care measures).
- Grade 4 neutropenia on 2 consecutive blood counts drawn at least 72 hours apart
- Grade 4 febrile neutropenia (ANC $< 1.0 \times 10^9$/L and fever ≥ 38.5° C), or grade ≥ 3 documented infection with ANC $< 1.0 \times 10^9$/L
- A platelet count < 25,000 /µL

The following hematological toxicities grade ≥3 will **not** be considered DLT:

- Grade ≥ 3 decrease in hemoglobin that can be corrected to at least 8.0g/dl (grade 2) by transfusion of red blood cells
- Grade ≥ 3 leukopenia in the absence of dose-limiting neutropenia

- Grade ≥ 3 lymphopenia

Any patient who experiences a DLT will be removed from the protocol. An exception would be if a patient on the protocol is benefiting from perifosine at dose level 1, 2 or 3, but experiences a DLT- the patient can continue on perifosine but at a lower dose level (if at dose level 3, then patient will be moved to dose level 2…etc)

9.4 Supportive Care Guidelines (all patients)

The guidelines in this section are suggestions. They may be altered according to the clinical discretion of the treating physician without being considered a protocol violation.

9.4.1 Antiemetic prophylaxis

All patients should have antiemetic medications prescribed and administered as needed, and adjusted during the cycle at the discretion of the treating investigator.

9.4.2 Diarrhea Management

All patients should be instructed to take loperamide at the earliest signs of diarrhea and/or abdominal cramping after beginning study medication. This can include (a) loose stool, (b) the occurrence of 1 to 2 more bowel movements than usual in 1 day, or (c) an unusually high volume of stool. Loperamide can be used at age appropriate doses. Additional antidiarrheal measures may be used at the discretion of the treating physician as warranted by the patient's condition.

9.4.3 G-CSF Administration

G-CSF may be used at the discretion of the treating physician

9.4.4 Other Concomitant Medications

Therapies considered necessary for the well-being of the patient may be given at the discretion of the investigator. Other concomitant medications should be avoided except for analgesics, chronic treatments for concomitant medical conditions, or agents required for life-threatening medical problems. All concomitant medications must be recorded.

9.4.5 Surgery
If surgical management is required for reasons not due to tumor progression, these procedures must be documented, including the indications for surgery, the surgical operative note and pathology report.

9.4.6 Corticosteroids should be used in the smallest dose to control symptoms of cerebral edema and mass effect, and discontinued if possible.

9.4.7 Anti-seizure medications should be used as indicated. However, only patients not taking EIAEDs are eligible to enroll on this trial. Patients who were previously on a non-EIAED and need to change anticonvulsants should be started on another non-EIAED if at all possible. No delays in treatment would be required. Patients who were previously on no anticonvulsants should be treated with non-EIAEDs if at all possible. If for unavoidable clinical reasons (severe allergies, toxicities etc.) a patient is started on an EIAED, he/she should immediately be started on another non-EIAED and the EIAED should be tapered and discontinued as quickly as possible. The patient may continue the current treatment dose while a non- EIAED is re-started because an EIAED will likely increase metabolism of perifosine, reducing rather than increasing any potential anti-tumor effect and therefore any efficacy bias introduced would be negative rather than positive.

Patients who were previously on a non-EIAED and need to permanently change anticonvulsant, but who cannot change to another non-EIAED may continue the current treatment dose for the next 2 weeks while an EIAED is started. FOLLOWING THIS PERIOD, THE SUBSEQUENT TREATMENT DOSE MUST BE DISCUSSED WITH THE PI OR CO-PI. Treatment may be allowed to continue if the patient is felt to be deriving clinical benefit.

EIAEDs and Non-EIAEDs

EIAEDs:
Carbamazepine (Tegretol, Tegretol XR, Carbatrol)
Oxcarbazepine (Trileptal)
Phenytoin (Dilantin, Phenytek)
Fosphenytoin (Cerebyx)
Phenobarbital
Primidone (Mysoline)

Non-EIAEDs:
Valproic acid (Depakote, Depakene)
Gabapentin (Neurontin)
Lamotrigine (Lamictal)

Topiramate (Topamax)
Tiagabine (Gabatril)
Zonisamide (Zonegran)
Levetiracetam (Keppra)
Clonazepam (Klonopin)
Clobazam (Frisium)

10.0 Evaluation during treatment/intervention

10.1 Clinical

History and physical examination at MSKCC will be performed as detailed in table below. Patients will have a follow up physical exam 1 week (± 2 days) after the patient starts treatment and approximately every first week of each subsequent cycle (four-week period).

Patients will maintain a weekly patient treatment diary to track doses of perifosine taken at home as well as any side effects experienced and return the diary to the clinic each visit.

10.2 Laboratory

Laboratory evaluation as described in table below.

10.3 Imaging Studies

Patients will undergo tumor assessment imaging studies approximately every 8 weeks. In general the follow-up scans should use the same imaging modality, but the investigator may use his/her best clinical judgment regarding the most appropriate scan (for example, in some cases PET may be substituted for MIBG).

10.4 Biological assay

15 unstained paraffin slides and/or 100mg of flash frozen tissue if available will be obtained from the surgery closest to initiation of this clinical trial. These will be used to evaluate molecular markers that could predict sensitivity to perifosine such as a PI3K/AKT activity, RAS/MAPK activity, proliferation rate, and somatic mutations of potential oncogenes or tumor suppressor genes in tumor cell DNA. These studies will be performed at an MSKCC core facility.

	Pre-Study	Wk 1	Wk 2	Wk 3	Wk 4	Wk 5	Wk 6	Wk 7	Wk 8	Wk 9	Wk 10	Wk 11	Wk 12	Wk 13	Off Study[c]
Perifosine administration[a]		X	X	X	X	X	X	X	X	X	X	X	X	X	
Informed consent	X														
Demographics	X														
Medical history	X														
Concurrent meds	X														
Physical exam	X		X			X				X				X[f]	
Vital signs	X		X			X				X				X[f]	
Height	X														
Weight	X		X			X				X				X[f]	
Performance status	X		X			X				X				X[f]	
CBC (w/diff, plts[f])	X		X			X				X				X[f]	
Serum chemistry[b]	X		X			X				X				X[d]	
Serum pregnancy test[e]	X														
Pharmacokinetic serum	X		X	X	X										
Adverse event evaluation	X	X --													X
Patient Diaries	X	X --													X
Tumor measurements	X	Tumor measurements are repeated every 8 weeks. Documentation must be provided for patients removed from study for progressive disease.													X[c]

a: Perifosine: Dose, route, schedule as assigned.
b: Albumin, alkaline phosphatase, total bilirubin, bicarbonate, BUN, calcium, chloride, creatinine, glucose, LDH, phosphorus, potassium, total protein, SGOT [AST], SGPT [ALT], sodium, uric acid, magnesium.
c: Off-study evaluation. It is preferable that two consecutive measurements taken to document progressive disease if the patient is removed from study for this reason.
d: Assessments to continue approximately every 12 weeks while patient is on study.
e: Within 14 days prior to initiation of therapy for women of childbearing potential only.
f: Assessments to continue approximately every 4 weeks while patient is on study.

Evaluations at weeks 2, 5 and approximately every 4 weeks thereafter have to be completed at MSKCC. Patients consented prior to March 16, 2011 are allowed to have their mandated evaluations performed at other centers as long as they are submitted to the PI in a timely fashion.

11.0 TOXICITIES/SIDE EFFECTS

The most common toxicities associated with perifosine are nausea, vomiting, and diarrhea. NCI CTC version 3.0 [34] will be used to grade all toxicity.

Below are some side-effects that have been reported in a variety of doses and schedules in adult trials (refer to table 1.3 for more details).

Likely side effects (> 20%)

- Diarrhea
- Nausea
- Vomiting
- Fatigue

Less Likely side effects (≤ 20%)

- Anemia
- Abdominal pain
- Constipation
- Flatulence
- Infection*
- Alkaline phosphatase increased
- Creatinine increased
- GGT increased
- Lymphocyte count decreased
- Weight loss
- Anorexia
- Dehydration
- Hyperglycemia
- Hypoalbuminemia
- Hypocalcemia
- Hypokalemia
- Hyponatremia
- Back pain
- Dyspnea
- Hiccups

* Infection includes all 75 sites of infection under the INFECTIONS AND INFESTATIONS SOC.

Also reported on Perifosine trials but with the relationship to Perifosine still undetermined:
CARDIAC DISORDERS - Atrial fibrillation; Cardiac arrest; Cardiac disorders - Other (cardiac valve disorder); Conduction disorder; Myocardial infarction; Palpitations; Pericardial effusion; Sinus bradycardia; Sinus tachycardia.
EAR AND LABYRINTH DISORDERS - Ear pain; Middle ear inflammation; Tinnitus
EYE DISORDERS - Blurred vision; Cataract; Conjunctivitis; Dry eye; Eye pain; Flashing lights; Keratitis; Photophobia; Watering eyes.
GASTROINTESTINAL DISORDERS - Abdominal distension; Ascites; Colitis; Colonic obstruction; Dry mouth; Dyspepsia; Dysphagia; Esophageal pain; Esophageal perforation; Esophageal stenosis; Esophagitis; Gastric hemorrhage; Gastritis; Gastrointestinal pain; Gingival pain; Hemorrhoids; Ileus; Lower gastrointestinal hemorrhage; Mucositis oral; Oral hemorrhage; Oral pain; Rectal hemorrhage; Rectal pain; Small intestinal obstruction; Stomach pain; Toothache; Upper gastrointestinal hemorrhage.
GENERAL DISORDERS AND ADMINISTRATION SITE CONDITIONS - Chills; Edema limbs; Fever; Flu like symptoms; Gait disturbance; General disorders and administration site conditions - Other (visceral edema); Localized edema; Non-cardiac chest pain; Pain.
HEPATOBILIARY DISORDERS - Hepatic failure; Hepatic pain.
IMMUNE SYSTEM DISORDERS - Allergic reaction.
INJURY, POISONING AND PROCEDURAL COMPLICATIONS - Arterial injury; Bruising; Fracture.
INVESTIGATIONS - Activated partial thromboplastin time prolonged; Alanine aminotransferase increased; Aspartate aminotransferase increased; Blood bilirubin increased; CPK increased; Carbon monoxide diffusing capacity decreased; Cholesterol high; INR increased; Investigations - Other (granulocytopenia); Lipase increased; Neutrophil count decreased; Platelet count decreased; Serum amylase increased; Weight gain; White blood cell decreased.
METABOLISM AND NUTRITION DISORDERS - Acidosis; Glucose intolerance; Hypercalcemia; Hyperkalemia; Hypernatremia; Hyperuricemia; Hypoglycemia; Hypomagnesemia; Hypophosphatemia; Metabolism and nutrition disorders - Other (blood bicarbonate decreased).
MUSCULOSKELETAL AND CONNECTIVE TISSUE DISORDERS - Arthralgia; Arthritis; Bone pain; Buttock pain; Chest wall pain; Generalized muscle weakness; Joint range of motion decreased; Muscle weakness left-sided; Muscle weakness lower limb; Muscle weakness upper limb; Musculoskeletal and connective tissue disorder - Other (upper extremity dysfunction); Myalgia; Myositis; Neck pain; Pain in extremity.
NEOPLASMS BENIGN, MALIGNANT AND UNSPECIFIED (incl cysts and polyps) - Neoplasms benign, malignant and unspecified (incl cysts and polyps) - Other (massive tumor hemorrhage); Tumor pain.
NERVOUS SYSTEM DISORDERS - Acoustic nerve disorder NOS; Ataxia; Cognitive disturbance; Depressed level of consciousness; Dizziness; Dysarthria; Dysgeusia; Extrapyramidal disorder; Facial nerve disorder; Headache; Intracranial hemorrhage; Memory

impairment; Neuralgia; Peripheral sensory neuropathy; Pyramidal tract syndrome; Seizure; Syncope; Tremor; Trigeminal nerve disorder; Vagus nerve disorder
PSYCHIATRIC DISORDERS - Agitation; Anxiety; Confusion; Depression; Insomnia; Libido decreased; Personality change; Psychosis.
RENAL AND URINARY DISORDERS - Acute kidney injury; Cystitis noninfective; Proteinuria; Urinary frequency; Urinary incontinence; Urinary retention; Urinary tract obstruction; Urinary tract pain
REPRODUCTIVE SYSTEM AND BREAST DISORDERS - Breast pain; Pelvic pain; Scrotal pain; Vaginal discharge; Vaginal hemorrhage
RESPIRATORY, THORACIC AND MEDIASTINAL DISORDERS - Allergic rhinitis; Atelectasis; Bronchial obstruction; Bronchospasm; Cough; Epistaxis; Hypoxia; Nasal congestion; Pharyngolaryngeal pain; Pleural effusion; Pleuritic pain; Pneumonitis; Pneumothorax; Voice alteration
SKIN AND SUBCUTANEOUS TISSUE DISORDERS - Alopecia; Dry skin; Hyperhidrosis; Pain of skin; Photosensitivity; Pruritus; Purpura; Rash acneiform; Rash maculo-papular; Skin atrophy; Skin hyperpigmentation; Skin hypopigmentation; Skin ulceration
VASCULAR DISORDERS - Flushing; Hot flashes; Hypertension; Hypotension; Superficial thrombophlebitis; Thromboembolic event

If hematologic toxicity grade 3 or higher occurs patients can be treated with appropriate growth factors.

12.0 CRITERIA FOR THERAPEUTIC RESPONSE/OUTCOME ASSESSMENT

This study is a phase I study and thus primarily a safety study of perifosine in a pediatric population with a variety of solid tumor histologies. Although objective responses will be analyzed as per RECIST criteria [34], this study will focus on determining an MTD, assessing whether PK levels correlate with objective responses, as well as determining whether any molecular features of the tumor can predict objective response.

Therapeutic response in patients with NB will be assessed using the International Neuroblastoma Response Criteria [35] which are shown below.
International Neuroblastoma Response Criteria:

- Complete remission/response: Complete disappearance of all evidence of disease.
- Very good partial remission/response: >90% decrease in volume of primary tumor and in all other markers of disease, except bone scan (if initially abnormal) stable or improved; BM must be free of disease
- Partial remission/response: 50 to 90% decrease in measurable, and no new, lesions, except bone scan stable or improved (if initially abnormal). Bones and BM: no more than 1 positive BM site allowed.
- Mixed response (MR): No new lesions; >50% reduction of any measurable lesion (primary or metastases) with <50% reduction in any other; <25% increase in any existing lesion.
- No response (NR; stable disease): <50% reduction in all tumor markers; no new

lesions; <25% increase in any existing lesion.
- Progressive disease (PD): Any new lesion; increase of any measurable lesion by >25%; previous negative marrow positive for tumor.

13.0 CRITERIA FOR REMOVAL FROM STUDY

All patients may continue therapy unless disease progression, death, or dose limiting toxicity is documented. The cause of death should be recorded.

The following events may be considered sufficient reason for discontinuing treatment with the study medication

- Serious toxicity due to the study drug graded according to the NCI Common Terminology Criteria for Adverse Events v3.0
- Conditions requiring therapeutic intervention not permitted by the protocol
- Unacceptable toxicity in the opinion of the patient or investigator even if not specifically defined elsewhere
- Personal preference by the patient for any reason
- Subject non-compliance with the defined treatment plan
- Medical or psychiatric illness or social environment which in the investigator's judgment renders the patient incapable of further therapy.
- Any other situation where, in the opinion of the investigator, continued participation in the study would not be in the best interest of the patient
- Pregnancy
- Disease progression

All reasons for discontinuation of treatment must be documented in the flow sheets.

14.0 BIOSTATISTICS

14.1 Dose escalation

This is a Phase I study designed to determine the maximum tolerated dose (MTD) of perifosine monotherapy for pediatric patients with recurrent or progressive solid tumors.

Patients will be treated in cohorts of size three to six and the dosage will be escalated if the clinical toxicity is acceptable. A patient is considered toxicity-free for the purpose of the trial if he/she completes the first cycle of therapy (28 days) without experiencing dose limiting toxicity (DLT). If therapy is discontinued during the first cycle for reasons other than toxicity (i.e.: tumors progression under therapy), an additional patient may be enrolled at that dose level to ensure adequate evaluation of toxicity. No within-patient escalation will be performed. Five dose levels of perifosine will be considered for escalation, and one dose will be considered for de-escalation. The precise dosing is depicted section 9.1 where each patient will be assigned to a dosing group according to their body surface area (four dosing groups).

Dose limiting toxicity (DLT) is defined as in section 9.3 and the design is constructed to minimize the chances of escalating the dose when the probability of DLT is high, and maximize the chance of escalating the dose when the probability of DLT is low. The dose escalation scheme is as follows:

1. If none of the initial three patients at a given dose level experience DLT, the next dose level will be studied.
2. If one of the initial three patients at a given dose level experiences DLT, three additional patients will be treated at the same dose level. Escalation will continue only if there has been no additional DLT observed.
3. If two or more patients experience DLT at a given dose, the previous dose will be declared the MTD. If this occurs at the starting dose, then the next three patients will be enrolled at dose level reserved for deescalation.
4. If only three patients were treated at a dose under consideration as MTD, an additional three patients will be treated at that level to confirm previous results.

The dose escalation-deescalation scheme provides the following probabilities of escalation based on the true chances of DLT at a specific dose level. One can see that the probability of escalation is higher as the toxicity risks are lower.

True Risk of Toxicity	10%	20%	30%	40%	50%	60%
Probability of Escalation	91%	71%	49%	31%	17%	8%

A minimum of 4 and a maximum of 36 patients will be required to complete the trial. With a planned accrual of one patient/month, the study should be completed within 36 months.

If the number of subjects who also have available biological assay permits, we will use three separate logistic models to determine whether molecular features of the tumor (elevated PI3K/AKT/mTOR signaling, elevated RAS/MAPK signaling and Cell cycle markers, respectively) predict the response to perifosine.

Using pharmacokinetics data collected at baseline and weekly during weeks 2-4, we will employ the GEE method to estimate the association between the probability of developing a DLT event and perifosine levels in the serum.

14.2 Neuroblastoma cohort

Following excellent safety profile observed at dose level #3, additional patients with neuroblastoma will be treated at dose level #3 for a preliminary evaluation of the efficacy of perifosine monotherapy against this cancer. Efficacy is measured in terms of response rate where response is either an objective response (CR, VGPR, PR, MR by the International

Neuroblastoma Response Criteria – see section 12.0) or progression free at 6 months. Note that a patient who has an objective response but progresses within 6 months will be considered a responder.

Response rate of 10% will be considered undesirable and 25% promising. The goal of the study is to assess whether there is an indication that the response rate is promising so that a larger Phase II or III study can be initiated. Thus we will use type II error rate of 10% for the study design.

In order to achieve our objective we obtained the minimum number of subjects necessary such that the probability of observing 0 or 1 responses is 10% if the true response rate is 25%. This number is 14 and decision rule is launch a Phase II or III if at least 2 patients respond. The probability of launching a Phase II or III study is 41.5%, 80.2% and 89.9% respectively when the true response rates are 10%, 20% and 25%.

The follow-up study can be designed to have 10% type I error rate and 90% power so that the combined type I error rate will be less than 5% and power over 80%.

If three or more of the 14 neuroblastoma patients experience DLT at dose level #3, we will revisit the MTD of the trial. The probability of revisiting the MTD is 16%, 55% or 84% for a true toxicity rate of 10%, 20%, or 30%, respectively.

The data analysis for the study will be performed by Dr. Oren Becher and his team at Duke University Medical Center.

15.0 RESEARCH PARTICIPANT REGISTRATION AND RANDOMIZATION PROCEDURES

15.1 Research Participant Registration

Confirm eligibility as defined in the section entitled Criteria for Patient/Subject Eligibility.

Obtain informed consent, by following procedures defined in section entitled Informed Consent Procedures.

During the registration process registering individuals will be required to complete a protocol specific Eligibility Checklist.

All participants must be registered through the Protocol Participant Registration (PPR) Office at Memorial Sloan-Kettering Cancer Center. PPR is available Monday through Friday from 8:30am – 5:30pm at 646-735-8000. Registrations must be submitted via the PPR Electronic Registration System (http://ppr/). The completed signature page of the written consent/RA or verbal script/RA, a completed Eligibility Checklist and other relevant documents must be uploaded via the PPR Electronic Registration System.

16.0 DATA MANAGEMENT ISSUES

A Research Study Assistant (RSA) will be assigned to the study. The responsibilities of the RSA include project compliance, data collection, abstraction and entry, data reporting, regulatory monitoring, problem resolution and prioritization, and coordinate the activities of the protocol study team.

The data collected for this study will be entered into a secure database. Source documentation will be available to support the computerized patient record.

16.1 Quality Assurance

Weekly registration reports will be generated to monitor patient accruals and completeness of registration data. Routine data quality reports will be generated to assess missing data and inconsistencies. Accrual rates and extext and accuracy of evaluations and follow-up will be monitored periodically throughout the study period and potential problems will be brought to the attention of the study team for discussion and action

Random-sample data quality and protocol compliance audits will be conducted by the study team, at a minimum of two times per year, more frequently if indicated.

16.2 Data and Safety Monitoring

The Data and Safety Monitoring (DSM) Plans at Memorial Sloan-Kettering Cancer Center were approved by the National Cancer Institute in September 2001. The plans address the new policies set forth by the NCI in the document entitled "Policy of the National Cancer Institute for Data and Safety Monitoring of Clinical Trials" which can be found at: http://www.cancer.gov/clinicaltrials/conducting/dsm-guidelines/page1. The DSM Plans at MSKCC were established and are monitored by the Office of Clinical Research. The MSKCC Data and Safety Monitoring Plans can be found on the MSKCC Intranet at: http://smskpsps9/dept/ocr/OCR%20Website%20Documents/Clinical%20Research%20Quality%20Assurance%20(CRQA)/MSKCC%20Data%20and%20Safety%20Monitoring%20Plan.pdf

There are several different mechanisms by which clinical trials are monitored for data, safety and quality. There are institutional processes in place for quality assurance (e.g., protocol monitoring, compliance and data verification audits, therapeutic response, and staff education on clinical research QA) and departmental procedures for quality control, plus there are two institutional committees that are responsible for monitoring the activities of our clinical trials programs. The committees: *Data and Safety Monitoring Committee (DSMC)* for Phase I and II clinical trials, and the *Data and Safety Monitoring Board (DSMB)* for Phase III clinical trials, report to the Center's Research Council and Institutional Review Board.

During the protocol development and review process, each protocol will be assessed for its level of risk and degree of monitoring required. Every type of protocol (e.g., NIH sponsored, in-house sponsored, industrial sponsored, NCI cooperative group, etc.) will be addressed and the monitoring procedures will be established at the time of protocol activation.

17.0 PROTECTION OF HUMAN SUBJECTS

17.1 Privacy

MSKCC's Privacy Office may allow the use and disclosure of protected health information pursuant to a completed and signed Research Authorization form. The use and disclosure of protected health information will be limited to the individuals described in the Research Authorization form. A Research Authorization form must be completed by the Principal Investigator and approved by the IRB and Privacy Board.

17.2 Serious Adverse Event (SAE) Reporting

Any SAE must be reported to the IRB as soon as possible but no later than 5 calendar days. The IRB requires a Clinical Research Database (CRDB) AE report to be emailed to sae@mskcc.org containing the following information:

Fields populated from the CRDB:

- Subject's name (generate the report with only initials if it will be sent outside of MSKCC)
- Medical record number
- Disease/histology (if applicable)
- Protocol number and title

Data needing to be entered:

- The date the adverse event occurred
- The adverse event
- Relationship of the adverse event to the treatment (drug, device, or intervention)
- If the AE was expected
- The severity of the AE
- The intervention
- Detailed text that includes the following information:
  - A explanation of how the AE was handled
  - A description of the subject's condition
  - Indication if the subject remains on the study
  - If an amendment will need to be made to the protocol and/or consent form

The PI's signature and the date it was signed are required on the completed report.

The CRDB AE report should be completed as above and the FDA assigned IND/IDE number written at the top of the report. If appropriate, the report will be forwarded to the FDA by the SAE staff through the IND Office.

17.2.1 SAE reporting to Aeterna Zentaris GmbH

All serious adverse events will also be reported to Aeterna Zentaris GmbH. This includes serious, related, labeled (expected) and serious, related, unlabeled (unexpected) adverse experiences.

All serious adverse events (SAEs) should be reported immediately to Aeterna Zentaris GmbH except for those SAEs that the protocol or other document (e.g., Investigator's Brochure) identifies as not needing immediate reporting. The investigator should also comply with the applicable regulatory requirement(s) related to the reporting of unexpected serious adverse drug reactions to the regulatory authority(ies) and the IRB/IEC.

For reported deaths, the investigator should supply Aeterna Zentaris GmbH and the IRB/IEC with any additional requested information (e.g., autopsy reports and terminal medical reports).

The investigator will complete the initial SAE information and email (drugsafety@aezsinc.com) or fax it to Æterna Zentaris at 1-800-767-3588 within 24 hours of becoming aware of the event. This information will constitute the initial report.

Relevant medical reports should also be provided as attachments. Aeterna Zentaris will review the information and obtain necessary (immediately required) documents or clarifications from the site.

Follow up SAE reports should be handled in the same fashion.

17.2.2 Relationship between the adverse event and the study drug

The Investigator must also assess the relationship of any adverse event to the use of study drug, based on available information, using the following guidelines:

Unlikely - no temporal association, or the cause of the event has been identified, or the drug cannot be implicated.
Possibly - temporal association, but other etiologies are likely to be the cause; however, involvement of the drug cannot be excluded.
Probably - temporal association, other etiologies are possible, but unlikely.
Definite- the adverse event is clearly related to the investigational agent.
Unrelated- the adverse event is clearly not related to the investigational agent.

17.2.3 Definition of Adverse Event

Adverse events are illnesses, signs, or symptoms that appear or worsen during the course of a study. Adverse events occurring in association with study drug administration, whether believed by the investigator to be related or unrelated to the drug should be recorded on the case report form. When the investigator is confident of the diagnosis, he should group together as a single illness all related signs, symptoms, and abnormal laboratory results, e.g. cough; rhinitis, and sneezing can be reported as "upper respiratory infection."

Abnormal laboratory results should be noted on the case report form if they are associated with an overdose, require or prolong inpatient hospitalization or are otherwise considered significant by the investigator.

17.2.4 Definition of Serious Adverse Event

A serious adverse event is one that meets any of the following criteria:

- life-threatening (all grade 4 events except myelosuppression, nausea, emesis, and hyperglycemia) or fatal
- substantial or permanent disability
- requires or prolongs inpatient hospitalization (this does not include hospitalization for elective treatment or emergency room visits)
- secondary malignancy
- a congenital anomaly in the offspring of a patient who received trial medication
- an event resulting from an overdose (overdoses without clinical sequelae are not serious adverse events)
- an event that may jeopardize the patient or may require intervention to prevent one of the outcomes listed above

18.0 INFORMED CONSENT PROCEDURES

Before protocol-specified procedures are carried out, consenting professionals will explain full details of the protocol and study procedures as well as the risks involved to participants prior to their inclusion in the study. Participants will also be informed that they are free to withdraw from the study at any time. All participants must sign an IRB/PB-approved consent form indicating their consent to participate. This consent form meets the requirements of the Code of Federal Regulations and the Institutional Review Board/Privacy Board of this Center. The consent form will include the following:

1. The nature and objectives, potential risks and benefits of the intended study.
2. The length of study and the likely follow-up required.

3. Alternatives to the proposed study. (This will include available standard and investigational therapies. In addition, patients will be offered an option of supportive care for therapeutic studies.)
4. The name of the investigator(s) responsible for the protocol.
5. The right of the participant to accept or refuse study interventions/interactions and to withdraw from participation at any time.

Before any protocol-specific procedures can be carried out, the consenting professional will fully explain the aspects of patient privacy concerning research specific information.

In addition to signing the IRB Informed Consent, all patients must agree to the Research Authorization component of the informed consent form.

Each participant and consenting professional will sign the consent form. The participant must receive a copy of the signed informed consent form.

19.0 reference(s)

1. Ries LAG, Smith MA, Gurney JG, Linet M, Tamra T, Young JL, et al. Cancer incidence and survival among children and adolescents: United States SEER Program 1975-1995, National Cancer Institute, SEER Program. NIH Pub. No. 99-4649. Bethesda, MD. 1999.

2. U.S. Cancer Statistics Working Group. United States Cancer Statistics: 1999-2002 Incidence and Mortality Web–based Report Version. Department of Health and Human Services, Centers for Disease Control and Prevention, and National Cancer Institute. Available at: http://www.cdc.gov/cancer/npcr/uscs/.

3. Athale UH, Stewart C, Kuttesch JF, Moghrabi A, W. M, Pratt C, Gajjar A, Heideman RL. Phase I study of combination topotecan and carboplatin in pediatric solid tumors. J Clin Oncol. 2002;20(1):88–95.

4. Adamson PC, Horowitz ME, Poplack DG. The child with recurrent solid tumor. Pediatr Clin North Am. 1991;38(2):489–504.

5. Hilgard, P., et al., D-21266, a new heterocyclic alkylphospholipid with antitumour activity. Eur J Cancer, 1997. 33(3): p. 442-6.

6. Arthur, G. and R. Bittman, The inhibition of cell signaling pathways by antitumor ether lipids. Biochim Biophys Acta, 1998. 1390(1): p. 85-102.

7. Ruiter, G.A., et al., Anti-cancer alkyl-lysophospholipids inhibit the phosphatidylinositol 3-kinase-Akt/PKB survival pathway. Anticancer Drugs, 2003. 14(2): p. 167-73.

8. Kondapaka, S.B., et al., Perifosine, a novel alkylphospholipid, inhibits protein kinase B activation. Mol Cancer Ther, 2003. 2(11): p. 1093-103.

9. Holland, E.C., et al., Combined activation of Ras and Akt in neural progenitors induces glioblastoma formation in mice. Nat Genet, 2000. 25(1): p. 55-7.

10. Opel D, Poremba C, Simon T, Debatin KM, Fulda S. Activation of AKT predicts poor outcome in neuroblastoma. Cancer Res 67:735-45.

11. Petricoin EF, Espina V, Araujo RP et al. Phosphoprotein pathway mapping: AKT/ mammalian target of rapamycin activation is negatively associated with childhood rhabdomyosarcoma survival. Cancer Res 67:3431-40.

12. Faury D, Nantel A, Dunn SE et al. Molecular profiling identifies prognostic subgroups of pediatric glioblastoma and shows increased YB-1 expression in tumors. J Clin Oncol 25: 1196-208

13. Hartmann W, Digon-Sontgerath B, Koch A, Waha A et al. PI3K/AKT signaling is activated in medulloblastoma cell proliferation and is associated with reduced expression of PTEN. Clin Cancer Res 2006:3019-27.

14. Chang, S.M., et al., Phase II study of CCI-779 in patients with recurrent glioblastoma multiforme. Invest New Drugs, 2005. 23: 357-61.

15. O'Reilly K, et al. mTOR inhibition induces upstream receptor tyrosine kinase signaling and activates Akt. Cancer Res 2006;66:1500-8.

16. Momota H., et al. Perifosine inhibits multiple signaling pathways in glial progenitors and cooperates with temozolomide to arrest cell proliferation in gliomas in vivo. Cancer Res. 2005 ;65:7429-35.

17. Mross, K., et al. Phase I study with weekly perifosine (D-21266) in patients with advanced cancer. in 25th National Cancer Congress of the German Cancer Society. 2000. Berlin: Journal of Cancer Research and Clinical Oncology.

18. Monga, M., et al. A phase 1 trial of oral perifosine in patients with refractory neoplasma. in American Society of Clinical Oncology. 2002: Journal of Clinical Oncology.

19. Van Ummerson, L., et al., A phase I trial of perifosine (NSC 639966) on a loading dose/maintenance dose schedule in patients with advanced cancer. Clin Cancer Res, 2004. 10(22): p. 7450-6.

20. Henderson, I.C., et al., A phase 1 study of weekly, divided dose perifosine in patients (pts) with non-small cell lung cancer (NSCLC),. J Clin Oncol (Proceedings, Amer Soc. Clin Res), 2006. 24(18S): p. A13063.

21. Crul, M., et al., Phase I and pharmacological study of daily oral administration of perifosine (D-21266) in patients with advanced solid tumours. Eur J Cancer, 2002. 38(12): p. 1615-21.

22. Argiris, A., et al., A phase II trial of perifosine, an oral alkylphospholipid, in recurrent or metastatic head and neck cancer. Cancer Biol Ther, 2006. 5(7): p. 766-70.

23. Posadas, E.M., et al., A phase II study of perifosine in androgen independent prostate cancer. Cancer Biol Ther, 2005. 4(10): p. 1133-7.

24. Bailey, H.H., et al., Phase II study of daily oral perifosine in patients with advanced soft tissue sarcoma. Cancer, 2006. 107(10): p. 2462-7.

25. Knowling, M., et al., A phase II study of perifosine (D-21226) in patients with previously untreated metastatic or locally advanced soft tissue sarcoma: A National Cancer Institute of Canada Clinical Trials Group trial. Invest New Drugs, 2006. 24(5): p. 435-9.

26. Ernst, D.S., et al., Phase II study of perifosine in previously untreated patients with metastatic melanoma. Invest New Drugs, 2005. 23(6): p. 569-75.

27. Leighl, N.B., et al., A Phase 2 study of perifosine in advanced or metastatic breast cancer. Breast Cancer Res Treat, 2007.

28. Hedley, M.J.M., et al., A Phase II trial of perifosineas second line therapy for advanced pancreatic cancer. A study of the Princess Margaret Hospital [PMH] Phase II Consortium. J Clin Oncol, 2005. Annual Meeting Proceedings.

29. Marsh, R.d.W., Lima, C. M. R., Levy, D. E., Mitchell, E. P., Rowland, K. M., Benson, A. B., A Phase II Trial of Perifosine in Locally Advanced, Unresectable, or Metastatic Pancreatic Adenocarcinoma. Am J Clin Oncol, 2007. 30(1): p. 26-31.

30. Vink, S.R., et al., Tumor and normal tissue pharmacokinetics of perifosine, an oral anti-cancer alkylphospholipid. Invest New Drugs, 2005. 23(4): p. 279-86.

31. Birch, R.e.a., Perifosine (P) as an active agent in the treatment of patients with advanced sarcoma. J Clin Oncol (Meeting Abstracts), 2007. 25(18 Supplement): p. 10059.

32. Chawla, S.P.e.a., A phase II study of AP23573 (an mTOR inhibitor) in patients (pts) with advanced sarcomas. Proc Am Soc Clin Oncol, 2005. 24: p. 9068.

33. Common Toxicity Criteria for Adverse Events v3.0 (CTCAE) http://www.accessdata.fda.gov/scripts/cder/onctools/toxicity.cfm. 2003, National Cancer Institute, Cancer Therapy Evaluation Program (CTEP).

34. Therasse P, Arbuck S, Eisenhauer E, et al. New guidelines to evaluate the response to treatment in solid tumors. European Organization for Research and Treatment of Cancer, National Cancer Institute of the United States, National Cancer Institute of Canada. J Natl Cancer Inst. 2000;92(3):205–216.

35. Brodeur GM, Pritchard J, Berthold F, et al. Revisions of the international criteria for neuroblastoma diagnosis, staging, and response to treatment. *J Clin Oncol* 1993;11(8):1466-1477.

20.0 APPENDICES

20.1 Karnofsky Performance Status
20.2 Lansky Score (for children)
20.3 Supportive Care Guidelines for Controlling Side Effects Associated with the Administration of Perifosine

20.1 Karnofsky Performance Status

Patient's performance status will be graded according to the following scale:

Karnofsky Performance Status

KPS 100 Normal; no complaints; no evidence of disease
KPS 90 Able to carry on normal activity; minor signs or symptoms of disease
KPS 80 Normal activity with effort; some sign or symptoms of disease
KPS 70 Cares for self; unable to carry on normal activity or do active work
KPS 60 Requires occasional assistance, but is able to care for most personal needs
KPS 50 Requires considerable assistance and frequent medical care
KPS 40 Disabled; requires special care and assistance
KPS 30 Severely disabled; hospitalization is indicated, although death not imminent
KPS 20 Very sick; hospitalization necessary; active support treatment is necessary
KPS 10 Moribund; fatal processes progressing rapidly
KPS 0 Dead

20.2 Lansky Score (for children)

Lansky Score	Performance Status
100	Fully active, normal
90	Minor restrictions in physically strenuous activity
80	Active, but tires more quickly
70	Both greater restriction of and less time spent in play activity
60	Up and around, but minimal active play; keeps busy with quieter activities
50	Gets dressed but lies around much of the day, no active play but able to participate in all quiet play and activities
40	Mostly in bed; participates in quiet activities
30	In bed; needs assistance even for quiet play
20	Often sleeping; play entirely limited to very passive activities
10	No play; does not get out of bed
0	Unresponsive

20.3 Supportive Care Guidelines for Controlling Side Effects Associated with the Administration of Perifosine

<u>**REMINDERS**</u>

Patients should take all medication with food, and not on an empty stomach.

NAUSEA AND VOMITING

<u>Loading dose schedule</u>

- 5HT3 antagonist—such as, but not limited to, ondansetron and granisetron—can be administered every 12 hours on the day of therapy and every 12 hours for 24 hours *after* the day of therapy.
- Dexamethasone in conjunction with the 5HT3 antagonist *unless otherwise contraindicated*.

If the above steps are ineffective, the frequency of the 5HT3 antagonist can be increased.

- If the nausea could be related to anticipation, the use of lorazepam can be initiated.
- Patients should have some medication on hand for breakthrough nausea—such as hydroxyzine.

<u>Daily dosing schedule</u>

- Patients should take medication with food and not on an empty stomach.
- Although the use of prophylactic antiemetics should not be necessary for patients on daily dosing, they should have antiemetics on hand—such as ondansetron or hydroxyzine—if they do encounter nausea and/or vomiting. If these antiemetics are not effective, the treating physician may escalate the antiemetics to the ones listed above for the loading dose.

DIARRHEA

For both Loading and Daily Dose Schedules
<u>At the first sign of change in stool or bowel function initiate recommendations</u>

- Imodium (age appropriate dosing) at the first onset of diarrhea.
- Repeat Imodium until diarrhea subsides for 12 hours.
- Patients can take Imodium during the night to minimize interruption of sleep.
- Drink plenty of fluids.
- Avoid foods and liquids that can aggravate diarrhea—such as dairy products, seeds, nuts, etc.

- The treating physician may use other antidiarrhea medications at his/her discretion.

GOUT AND/OR HYPERURICEMIA

For both Loading and Daily Dose Schedules

- All patients should be asked about the possible prior history of gout or hyperuricemia.
- Patients with prior history of gout or elevated uric acid levels should receive allopurinol by mouth daily for prophylaxis.
- The treating physician may increase the dose of allopurinol (age appropriate dosing) or change medications at his/her discretion as warranted by the individual patient's status.

NOTE ABOUT THIS INFORMATION

- Treating physicians may use other agents at their discretion.
- These recommendations are based on current information obtained from clinical studies—that have either been completed or are underway—examining the efficacy and safety of perifosine.
